# Elicitation of Robust Tier 2 Neutralizing Antibody Responses in Nonhuman Primates by HIV Envelope Trimer Immunization Using Optimized Approaches

**DOI:** 10.1016/j.immuni.2017.05.007

**Published:** 2017-06-20

**Authors:** Matthias Pauthner, Colin Havenar-Daughton, Devin Sok, Joseph P. Nkolola, Raiza Bastidas, Archana V. Boopathy, Diane G. Carnathan, Abishek Chandrashekar, Kimberly M. Cirelli, Christopher A. Cottrell, Alexey M. Eroshkin, Javier Guenaga, Kirti Kaushik, Daniel W. Kulp, Jinyan Liu, Laura E. McCoy, Aaron L. Oom, Gabriel Ozorowski, Kai W. Post, Shailendra K. Sharma, Jon M. Steichen, Steven W. de Taeye, Talar Tokatlian, Alba Torrents de la Peña, Salvatore T. Butera, Celia C. LaBranche, David C. Montefiori, Guido Silvestri, Ian A. Wilson, Darrell J. Irvine, Rogier W. Sanders, William R. Schief, Andrew B. Ward, Richard T. Wyatt, Dan H. Barouch, Shane Crotty, Dennis R. Burton

**Affiliations:** 1 Department of Immunology and Microbiology, The Scripps Research Institute, La Jolla, CA 92037, USA; 2 Center for HIV/AIDS Vaccine Immunology and Immunogen Discovery, The Scripps Research Institute, La Jolla, CA 92037, USA; 3 IAVI Neutralizing Antibody Center and the Collaboration for AIDS Vaccine Discovery, The Scripps Research Institute, La Jolla, CA 92037, USA; 4 Division of Vaccine Discovery, La Jolla Institute for Allergy and Immunology, La Jolla, CA 92037, USA; 5 International AIDS Vaccine Initiative, New York, NY 10004, USA; 6 Center for Virology and Vaccine Research, Beth Israel Deaconess Medical Center, Harvard Medical School, Boston, MA 02215, USA; 7 Department of Microbiology and Immunology, Weill Medical College of Cornell University, New York, NY 10065, USA; 8 Department of Integrative Structural and Computational Biology, The Scripps Research Institute, La Jolla, CA 92037, USA; 9 Bioinformatics Core, Sanford Burnham Prebys Medical Discovery Institute, La Jolla, CA 92037, USA; 10 Koch Institute for Integrative Cancer Research, Massachusetts Institute of Technology, Cambridge, MA 02139, USA; 11 Department of Medical Microbiology, Academic Medical Center, University of Amsterdam, 1105 AZ Amsterdam, the Netherlands; 12 Ragon Institute of Massachusetts General Hospital, Massachusetts Institute of Technology, and Harvard University, Cambridge, MA 02139, USA; 13 Howard Hughes Medical Institute, Chevy Chase, MD 20815, USA; 14 Departments of Biological Engineering and Materials Science & Engineering, Massachusetts Institute of Technology, Cambridge, MA 02139, USA; 15 Department of Surgery, Duke University Medical Center, Durham, NC 27710, USA; 16 Skaggs Institute for Chemical Biology, The Scripps Research Institute, La Jolla, CA 92037, USA; 17 Division of Infectious Diseases, Department of Medicine, University of California, San Diego, La Jolla, CA 92037, USA; 18 Yerkes National Primate Research Center, Emory University, Atlanta, GA 30322, USA; 19 Emory Vaccine Center, Emory University School of Medicine, Atlanta, GA 30322, USA; 20 Division of Infection & Immunity, University College London, London WC1E 6BT, UK; 21 University of California, San Diego, La Jolla, CA 92093, USA

**Keywords:** HIV vaccine, nonhuman primates, rhesus macaques, BG505, SOSIP, NFL, GC B cells, protein design, Tfh cells, germinal centers

## Abstract

The development of stabilized recombinant HIV envelope trimers that mimic the virion surface molecule has increased enthusiasm for a neutralizing antibody (nAb)-based HIV vaccine. However, there is limited experience with recombinant trimers as immunogens in nonhuman primates, which are typically used as a model for humans. Here, we tested multiple immunogens and immunization strategies head-to-head to determine their impact on the quantity, quality, and kinetics of autologous tier 2 nAb development. A bilateral, adjuvanted, subcutaneous immunization protocol induced reproducible tier 2 nAb responses after only two immunizations 8 weeks apart, and these were further enhanced by a third immunization with BG505 SOSIP trimer. We identified immunogens that minimized non-neutralizing V3 responses and demonstrated that continuous immunogen delivery could enhance nAb responses. nAb responses were strongly associated with germinal center reactions, as assessed by lymph node fine needle aspiration. This study provides a framework for preclinical and clinical vaccine studies targeting nAb elicitation.

## Highlights

• Immunization protocols for rapid and consistent generation of autologous tier 2 nAbs
• Germinal center responses predict and correlate with HIV nAbs after immunization
• Env protein design curtails responses to the non-neutralizing V3-loop epitope
• Subcutaneous and extended immunogen delivery enhances nAb generation

## Graphical Abstract

**Figure:**
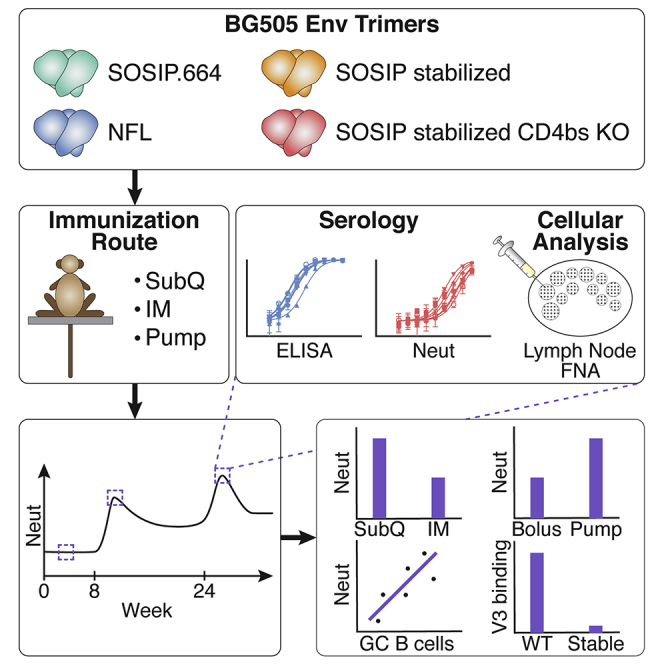

## Teaser

There is limited experience with recombinant Env trimer immunogens in nonhuman primates. Pauthner et al. compare multiple Env trimer designs and immunization strategies for generating HIV neutralizing antibodies. They identify protocols for rapid and consistent generation of tier 2 nAbs, providing a framework for future pre-clinical and clinical vaccine studies.

## Introduction

Successful vaccines to viral pathogens rely heavily on the induction of neutralizing antibody (nAb) responses for host protection (Plotkin, 2010). However, the induction of nAbs to circulating HIV (so-called tier 2 viruses) through immunization has proven very difficult. The surface HIV envelope (Env) spike, which consists of a heterotrimer of composition (gp120)_3_(gp41)_3_, is the sole target of HIV nAbs. All human HIV vaccine trials to date have failed to induce tier 2 nAbs (Haynes et al., 2012, Mascola and Montefiori, 2010). These trials mostly utilized monomeric Env gp120 or poor gp140 mimics of native Env spikes, which resulted in the generation of non-neutralizing or tier 1 nAbs only. The latter neutralize only lab-adapted and very easy-to-neutralize HIV strains, which are not representative of most viruses circulating in humans (Mascola et al., 2005). The failure to induce tier 2 nAb responses has been associated with differences between the presentation of critical epitopes on the immunogens used and their presentation on the native Env spike. The generation of molecules that more faithfully mimic the spike, particularly the SOSIP trimer (Binley et al., 2000, Sanders et al., 2002, Sanders and Moore, 2017, Sanders et al., 2013), has opened up new opportunities for the induction of tier 2 nAbs. Indeed, native-like Env trimers have successfully induced tier 2 nAbs in small animal models (de Taeye et al., 2016, Feng et al., 2016, McCoy et al., 2016, Sanders et al., 2015) and less reproducibly in nonhuman primates (NHPs) (Havenar-Daughton et al., 2016, Sanders et al., 2015).

NHPs, and specifically rhesus macaques (RMs), are often argued to be the most appropriate pre-clinical model for HIV vaccine studies because of the close genetic relatedness of NHPs to humans. Three RM studies using trimeric Env immunogens reported the induction of autologous tier 2 nAbs (Havenar-Daughton et al., 2016, Hessell et al., 2016, Sanders et al., 2015), and two of these studies used SOSIP trimer designs (Havenar-Daughton et al., 2016, Sanders et al., 2015). However, there have been concerns about the limitations of such results. Tier 2 nAb titers were primarily reported after 6–12 months of immunizations, and titers were relatively low. Most worrisome was the observation that only a fraction of the monkeys developed nAbs, raising concerns about whether Env trimers will elicit tier 2 nAbs in humans (Havenar-Daughton et al., 2017, Sanders and Moore, 2017).

To optimize the induction of autologous tier 2 nAb responses by native-like trimer immunizations, we investigated a number of parameters, including immunization route, dose, and timing of immunizations. We studied two trimer platforms, the SOSIP and native-flexible linker (NFL) platforms (Sharma et al., 2015). We investigated the effects of additional stabilizing mutations applied to the SOSIP platform and the effects of continuous and bolus immunization.

In all cases, we focused on the analysis of tier 2 nAb and non-neutralizing Ab responses, together with germinal center (GC) responses, in the draining lymph nodes (LNs). NAb development requires affinity maturation, which takes place in GCs under the control of GC T follicular helper (Tfh) cells (Crotty, 2014, Victora and Nussenzweig, 2012). GC activity after booster immunizations has been associated with nAb development in BG505 SOSIP immunized RMs (Havenar-Daughton et al., 2016). Thus, modulating GC B and Tfh cell quantities and qualities could help guide optimization of HIV nAb induction by immunization.

In summary, we tested multiple stabilized, trimeric Env immunogens and immunization strategies head-to-head to evaluate their impact on the quantity, quality, and kinetics of tier 2 nAb development. We found that unlike intramuscular (i.m.) injections, subcutaneous (s.c.) immunizations reliably induced nAb titers in NHPs and that longer intervals between prime and booster immunizations increased GC B cell frequencies. GC B frequencies predicted and correlated with nAb development in NHPs, and their expansion should thus be targeted by vaccination strategies. We showed that stabilizing mutations in the SOSIP immunogen can lead to lower HIV V3-loop responses and that continuous immunization can induce notably stronger nAb responses than bolus immunization. This study provides a framework for preclinical and clinical vaccine studies targeting the development of HIV nAbs.

## Results

### Induction of Potent nAb Titers after Two Immunizations with Env Trimers

**Figure 1:**
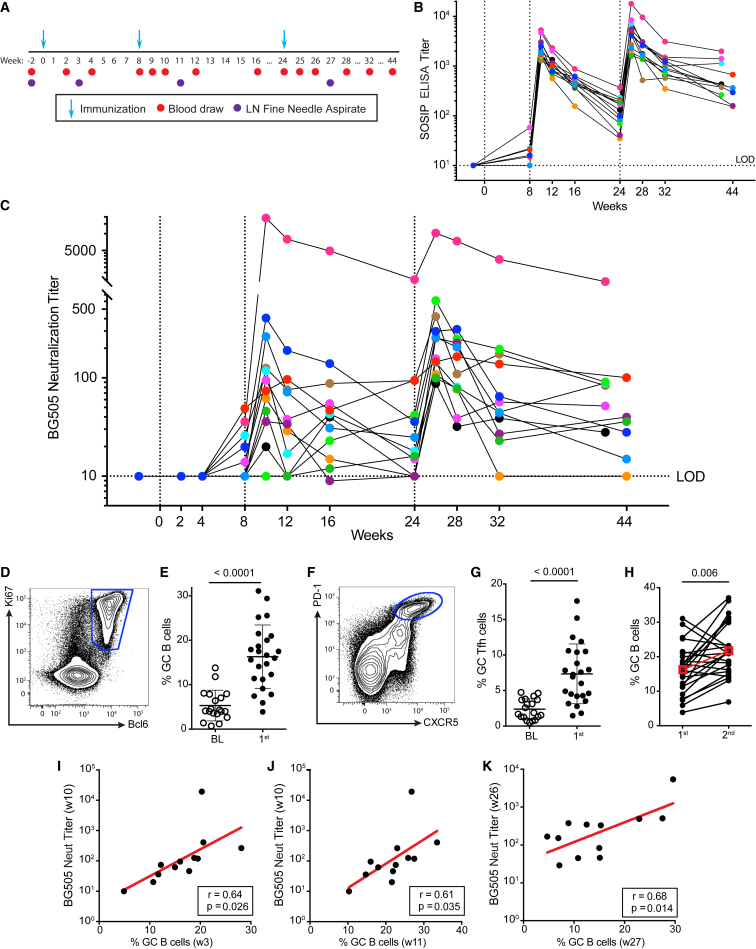
Induction of Potent HIV nAb Titers after Two Immunizations with Env Trimers and Correlations with GC B Cell frequency (A) 0-8-24 week immunization schedule and sampling. (B) BG505 SOSIP EC_50_ binding IgG titers of BG505-SOSIP.664-immunized RMs (n = 12). Colors indicate individual animals. Vertical dotted lines indicate immunization time points. LOD, limit of detection. (C) BG505 neutralization IC_50_ titers of BG505-SOSIP.664-immunized RMs over the course of the immunizations (n = 12). Colors are as in (B). (D) Flow-cytometry gating of GC B cells, gated on CD20^+^ B cells. (E) GC B cell frequencies at baseline (BL) and after the first immunization. Points represent individual LNs (n = 24). (F) Flow-cytometry gating of GC Tfh cells, gated on CD3^+^ CD4^+^ T cells. (G) GC Tfh cell frequencies at baseline (BL) and after the first immunization (n = 24). (H) GC B cell frequencies after the first and second immunizations. LNs sampled at both time points are connected by a black line. Red symbols indicate means. (I) GC B cell frequencies (mean of two draining LNs per animal) after the first immunization (week 3) predicts nAb titers after the second immunization (week 10). Red line shows linear regression (n = 12). (J and K) GC B cell frequencies correlate with nAb titers after the second (J) and third (K) immunizations (n = 12). All cell-frequency data represent the mean and SD. See also Figure S1.

To try to improve the consistency and magnitude of BG505 SOSIP.664 nAb responses, we evaluated a modified immunization protocol designed to boost and prolong GC activity between immunizations. In a previous study using BG505 SOSIP.v5.2 in RMs, elevated frequencies of GC B cells were most likely present for at least 6 weeks after the first immunization. However, administration of the second immunization at the 6 week time point resulted in weaker GC responses than the first immunization (Havenar-Daughton et al., 2016). Therefore, we extended the initial immunization interval from 6 to 8 weeks. A third immunization was scheduled at week 24, consistent with conventional human vaccine schedules (Figure 1A). Twelve RMs were each immunized with BG505 SOSIP.664 (100 μg) formulated with ISCOMATRIX adjuvant, and each dose was split between two immunization sites. ISCOMATRIX is a strong immunostimulatory complex (Sun et al., 2009) that does not disturb the trimer structure (Sanders et al., 2015) and has previously been used to stimulate tier 2 nAb responses (Havenar-Daughton et al., 2016, Sanders et al., 2015). Bilateral injections were used to potentially increase recruitment of rare antigen-specific B cells and CD4^+^ T cells. All animals developed high BG505 SOSIP binding IgG titers (>1:1,000) after the second immunization (Figure 1B). After the third immunization at week 24, BG505 SOSIP binding IgG titers peaked with titers ranging from 1:2,000 to 1:20,000 (Figure 1B).

To assess the development of autologous nAb titers, we tested sera for neutralization of BG505 pseudovirus by using TZM-bl cell neutralization assays (Figure 1C). At week 10, after only two immunizations, 11/12 animals developed nAb responses (1:140 geometric mean titer [GMT]). One animal, 12-084, developed an exceptionally strong nAb titer of 1:20,000. Tier 1 nAb titers (SF162 and MW965 pseudoviruses) were also tested and found to be uniformly high, in the range of 1:1,000–1:10,000 (Figure S1A). Sera from all animals were also tested for neutralization in the Montefiori laboratory at Duke University, and nAb titers correlated (p < 0.0001, r = 0.87; Figure S1B). BG505 nAb titers were boosted after the third immunization (1:209 GMT; Figure 1C). Importantly, 100% of RMs responded with a robust titer of approximately 1:100 or higher. No correlation was found between BG505 nAb titers and BG505 SOSIP or V3-peptide ELISA binding immunoglobulin G (IgG) titers (Figures S1C and S1D). There was, however, a correlation between tier 1 nAb titers and V3-peptide ELISA binding titers, as previously observed (Havenar-Daughton et al., 2016, Sanders et al., 2015). Thus, this modified BG505 SOSIP immunization protocol substantially improved response rates, response kinetics, and peak autologous HIV neutralization titers in RMs over those from earlier studies. These HIV tier 2 nAb responses are the strongest, earliest, and most consistent HIV tier 2 nAb responses reported to date after protein, DNA, or vector immunization of monkeys.

### Early GC Activity Predicts nAb Development

To investigate the underlying immunological mechanisms contributing to consistent and rapid nAb titer development, we performed LN fine needle aspirates (FNAs) after each immunization. GC B cell frequencies in the draining LNs were significantly increased after the first immunization (Figures 1D, 1E, and S1E), as were GC Tfh cell frequencies (Figures 1F, 1G, and S1E). GC B and Tfh cell frequencies were significantly higher than baseline values after each of the three immunizations (Figures S1F and S1G) and were correlated (r = 0.71, p < 0.0001; Figure S1H). In contrast to the previously examined 6 week booster interval, GC B cell frequencies after the second immunization at week 8 were significantly higher than after the first immunization (Figure 1H). In the previous study, GC responses partially correlated with nAb development (Havenar-Daughton et al., 2016), which led us to hypothesize that early GC responses to the first immunization in this study might predict the development of BG505 nAbs. Indeed, GC B cell frequencies 3 weeks after the first immunization predicted rapid autologous BG505 nAb titers at week 10 (r = 0.64, p = 0.026; Figure 1I). In addition, post-second-immunization GC B cells correlated with post-second-immunization nAb titers (r = 0.61, p = 0.035; Figure 1J), and post-third-immunization GC B cells correlated with post-third-immunization nAb titers (r = 0.68, p = 0.014; Figure 1K). In contrast, GC B cell frequency did not correlate with BG505 SOSIP ELISA binding IgG titers at any time point (Figure S1I) or tier 1 nAb titers (Figure S1J). In sum, GC B cell frequencies in the draining LNs predicted and correlated with the magnitude of nAb development throughout the immunization regimen, highlighting the importance of generating strong GC responses.

### Subcutaneous Immunizations Induce Stronger nAb Responses Than Intramuscular Immunizations

**Figure 2:**
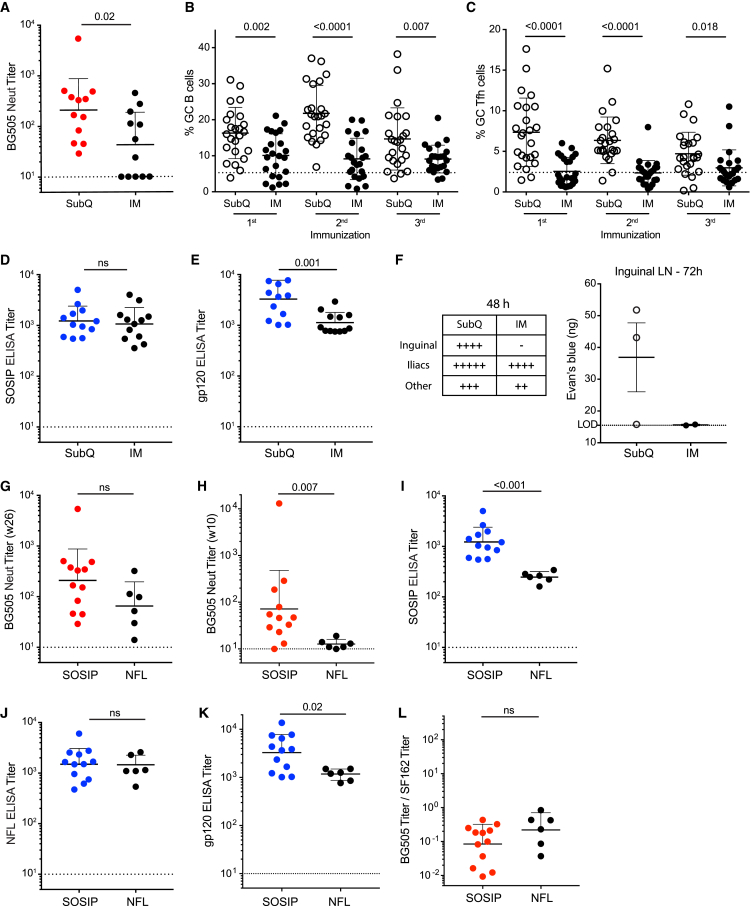
Subcutaneous Immunizations Induce Stronger Autologous nAb Responses Than Intramuscular Immunizations SOSIP.664 and NFL Env trimers have similar immunogenic properties. (A–F) Comparison of subcutaneous (s.c. or SubQ) and intramuscular (i.m. or IM) immunization routes with BG505 SOSIP.664 as immunogen. (A) BG505 nAb titers after BG505 SOSIP.664 s.c. or i.m. immunization (week 26; n = 12 animals per group). (B and C) GC B cell frequencies (B) and GC Tfh cell frequencies (C) 3 weeks after the first, second, and third s.c. or i.m. immunizations (n = 24 LNs per group). (D and E) BG505 SOSIP (D) and BG505 gp120 (E) IgG binding titers (week 26; n = 12). (F) Evans Blue dye drainage to LNs at 48 hr was scored by visual inspection (left). Dye accumulation in LNs at 72 hr was quantified by dye extraction (right). (G–K) Ab responses in RMs immunized with BG505 SOSIP.664 or BG505 NFL via s.c. injection. (G and H) BG505 nAb titers in BG505-SOSIP.664- and BG505-NFL-immunized RMs (n = 12 and 6, respectively) 2 weeks after the third (G) or second (H) immunization. (I–K) BG505 SOSIP (I), BG505 NFL (J), and BG505 gp120 (K) IgG binding titers 2 weeks after the third immunization (week 26; n = 6 or 12). (L) Ratio of BG505 to SF162 nAb titers 2 weeks after the third immunization (week 26; n = 6 or 12). All nAb titer and ELISA binding Ab data represent geometric mean titers with geometric SD. All cell-frequency data represent the mean and SD. The Evans blue quantification shows mean with SEM. See also Figure S2.

To evaluate the contribution of the route of immunization to the development of consistent and rapid HIV nAb titers, we compared RMs immunized by either s.c. or i.m. injection with equal doses of BG505 SOSIP.664 (100 μg) on the same schedule (Figure 1A). Peak nAb titers were significantly lower after i.m. immunization than after s.c. immunization (p = 0.02; Figures 2A and S2A). Only 7 of 12 animals immunized via i.m. injection developed a detectable nAb response, similar to the first BG505 SOSIP.664 RM immunization study, which used the i.m. route (Sanders et al., 2015). Lower GC B cell frequencies (p ≤ 0.007; Figure 2B) and GC Tfh cell frequencies (Figure 2C) were observed after i.m. immunizations than after s.c. immunizations.

We hypothesized that the difference between s.c. and i.m. GC and nAb responses could have arisen from lower availability of intact trimers for B cell recognition in the draining LNs after i.m. immunizations than after s.c. immunization. SOSIP binding titers were not significantly different between the i.m. and s.c. groups (Figure 2D), whereas both gp120 (Figure 2E) and V3-peptide Ab binding titers were lower in the i.m. group (Figure S2B), arguing against trimer decay as the underlying cause. We therefore examined LN drainage after i.m. or s.c. immunization in a separate experiment by using the trackable dye Evans Blue. The s.c. immunizations resulted in more rapid drainage to the inguinal LNs (48 hr; Figure 2F) and increased dye accumulation over time (72 hr; Figure 2F). The lymphatic drainage data suggest that more trimer immunogen reaches LN B cells after s.c. immunization, potentially increasing recruitment of antigen-specific B and CD4^+^ T cells and subsequent affinity maturation to improve nAb generation. In sum, s.c. immunization resulted in stronger autologous nAb responses than i.m. immunization.

### NFL Trimers Induce Approximately Equivalent but Slower nAb Responses Than SOSIP Trimers

NFL trimers have similar structural and antigenic properties to SOSIP trimers (Guenaga et al., 2015, Sharma et al., 2015), but an in vivo comparison of both platforms in RMs is lacking. We immunized six RMs with 100 μg of BG505 NFL trimers in parallel with RMs immunized with BG505 SOSIP.664 (Figure 1A). NFL- and SOSIP-immunized RMs developed similar peak nAb titers, although there was a trend toward somewhat lower titers for NFL (SOSIP = 1:209 GMT versus NFL = 1:65 GMT, p = 0.1; Figure 2G). NFL-induced nAb titers were statistically lower after the second immunization (Figure 2H). ELISA binding IgG titers were lower for BG505-NFL-immunized animals than for SOSIP-immunized animals when BG505 SOSIP was used as the ELISA antigen (Figure 2I), but they were similar when BG505 NFL was used as the antigen (Figure 2J). The differential ELISA data could indicate subtle differences between the SOSIP and NFL trimer bases or that overall structure and glycosylation influence immunogenicity. Of note, in contrast to BG505 SOSIP, the BG505 NFL design included a His-tag, which elicited an anti-His antibody (Ab) response (Figure S2C). BG505 gp120 binding titers, V3-peptide binding titers, and tier 1 nAb titers were also significantly lower in NFL-immunized animals (Figures 2K, S2D, and S2E). Ratios of tier 2 to tier 1 nAb titers were similar in NFL- and SOSIP-immunized animals (Figure 2L). GC B cell and GC Tfh cell frequencies were comparable across all measured time points (Figures S2F and S2G). Overall, BG505 NFL and SOSIP trimer constructs induced similar nAb and GC responses.

### Trimer Stabilization Strategies Can Reduce V3-Loop Antigenicity

Although BG505 SOSIP.664 trimers have generally excellent antigenic properties (Sanders et al., 2013), there is evidence for some flexibility of the molecule and exposure of regions, such as the V3 loop tip, that are not exposed on virion surface trimers (de Taeye et al., 2015). Indeed, SOSIP immunization has consistently produced relatively strong non-neutralizing anti-V3 responses that are not useful in a vaccine context and could be distracting (Mascola and Montefiori, 2010, Sanders et al., 2015). Therefore, we investigated the immunogenicity of four constructs designed to further stabilize the SOSIP molecule in general and to reduce the exposure of the V3 loop in particular (de Taeye et al., 2015, Guenaga et al., 2015, Steichen et al., 2016).

**Figure 3:**
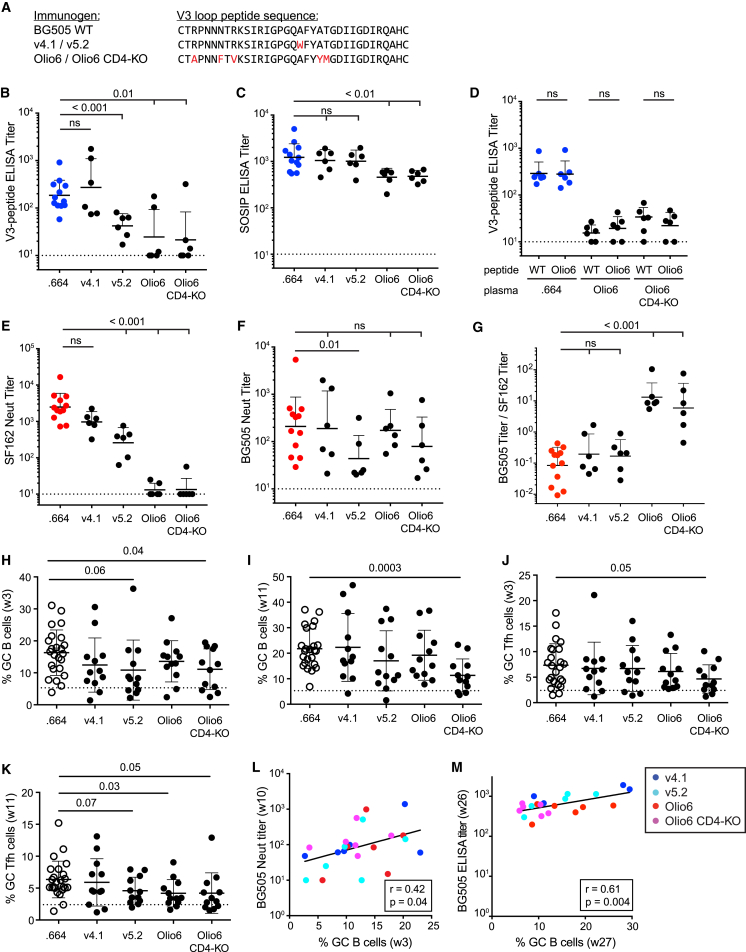
Trimer Stabilization Strategies Can Reduce V3-Loop Immunogenicity Modified BG505 SOSIP and BG505 Olio6 constructs were compared with the BG505 SOSIP.664 immunogen. Immunogens are abbreviated as follows: BG505 SOSIP.664, .664 or BG505 WT; BG505 SOSIP.v4.1, v4.1; BG505 SOSIP.v5.2, v5.2; BG505 SOSIP Olio6, Olio6; and BG505 SOSIP Olio6 CD4-KO, Olio6 CD4-KO. Dotted lines indicate LOD. (A) V3-loop protein sequence of BG505 WT, v4.1, v5.2, Olio6, and Olio6 CD4-KO. Changes are in red. (B) BG505 V3-loop peptide binding IgG titers 2 weeks after the third immunization (week 26; n = 6 or 12). (C) BG505 SOSIP binding IgG titers 2 weeks after the third immunization (week 26; n = 6 or 12). (D) Cross-binding analysis of Olio6 and SOSIP.664 (WT) V3-loop peptides (week 26; n = 6). See Figure 3A. (E–G) Tier 1 SF162 nAb titers (E), BG505 nAb titers (F), and their ratio (G) 2 weeks after the third immunization (week 26; n = 6 or 12). (H and I) GC B cell frequencies after the first (H) and second (I) immunizations (n = 24 or 12). (J and K) GC Tfh cell frequencies after the first (J) and second (K) immunizations (n = 24 or 12). (L) Correlation between GC B cell frequency and BG505 nAb titers. Animals are color coded by immunogen (n = 24). (M) Correlation between GC B cell frequency and BG505 SOSIP binding IgG titers (n = 24). All nAb titer and ELISA binding Ab data represent geometric mean titers with geometric SD. All cell-frequency data represent the mean and SD. See also Figure S3.

Groups of six RMs were immunized with 100 μg of BG505 SOSIP.v4.1 (de Taeye et al., 2015), BG505 SOSIP.v5.2 (A.T.d.l.P. et al., unpublished data; Havenar-Daughton et al., 2016), BG505 Olio6, or BG505 Olio6 CD4-KO (D.W.K. et al., unpublished data) in parallel with RMs immunized with BG505 SOSIP.664 after the same immunization scheme as in Figure 1A. Because a major focus of these four modified Env trimer designs was V3-loop stabilization and sequestration (Figure 3A), V3-peptide and gp120 binding IgG titers were compared. Three of the modified SOSIP constructs, but not SOSIP v4.1, elicited significantly lower V3-peptide ELISA binding titers than the wild-type (WT) SOSIP.664 molecule (Figure 3B). This pattern was repeated for gp120 binding IgG titers (Figure S3A), consistent with the notion that V3 reactivity is a major contributor to gp120 binding. Only the Olio6 constructs showed somewhat reduced SOSIP ELISA binding titers (Figure 3C), consistent with the finding that these constructs had the highest reduction in V3 Ab titers, which were detected in SOSIP ELISAs (Figure S3B). To exclude the possibility that the mutated V3-loop in Olio6 trimers elicits antibodies that do not cross-react with BG505 WT V3-loop residues, we compared V3 binding Ab titers with both WT and Olio6 V3-loop peptides for all groups and found no differences (Figure 3D). We separately confirmed WT and Olio6 V3-peptide antigenicity by comparing a panel of six well-characterized V3-loop Abs in peptide-binding ELISAs and found only one epitope to be slightly altered (Figure S3C), consistent with the extensive cross-reactivity of anti-V3-loop responses across HIV strains. As expected, ratios of SOSIP to V3-peptide binding Ab were increased for the three constructs that showed less V3-peptide binding than SOSIP.664 (Figure S3D). Further, ratios of BG505 nAb to V3-peptide binding Ab were increased for both Olio6 immunogens (Figure S3E), again consistent with the strong redirection of the Ab response away from the non-neutralizing V3-loop epitope.

We then examined induction of tier 1 nAb titers, which are driven most prominently by V3-loop-tip-directed Abs (Figures S3F and S3G). Compared with titers from when WT SOSIP.664 was used as the immunogen, tier 1 SF162 titers were similar in SOSIP.v4.1-immunized animals, reduced in SOSIP.v5.2-immunized animals, and almost completely abrogated in animals immunized with Olio6 and Olio6 CD4-KO (Figure 3E). In sum, V3-loop stabilization of the BG505 immunogen was successful at reducing the immunogenicity of the V3 epitope in immunized RMs for three of the four designs tested, and the Olio6 designs proved most effective.

Despite the reductions in V3 responses, we observed no differences in absolute tier 2 nAb responses between the modified constructs and WT SOSIP.664 (Figure 3F). Surprisingly, nAb titers were somewhat but significantly reduced for SOSIP.v5.2. However, both Olio6 and Olio6 CD4-KO induced 10- to 20-fold greater ratios of tier 2 to tier 1 nAb titers than SOSIP.664 (Figure 3G). The lack of enhanced nAb responses could arise because reduction or elimination of a single immunodominant non-neutralizing epitope site does not sufficiently shift the immunodominance hierarchy (Havenar-Daughton et al., 2017).

GC B cell and GC Tfh cell responses were compared between RMs immunized with different Env trimer designs. Olio6 CD4-KO generated significantly lower frequencies of GC B cells than did SOSIP.664 after the first and second immunizations (Figures 3H and 3I). GC Tfh cell frequencies were also significantly reduced in Olio6 CD4-KO groups after both the first and second immunizations (Figures 3J and 3K). The other stabilized immunogens followed a similar trend. The reduced GC B cell and Tfh cell frequencies could be explained by diminished V3-loop- and gp120-specific (rather than trimer-specific) B cells in the GCs. GC B cell frequencies correlated with BG505 nAb titers (Figures 3L and S3H). Unlike GC B cells in BG505-SOSIP.664-immunized animals, GC B cells also correlated with BG505 SOSIP binding titers (r = 0.61, p = 0.004; Figure 3M) after immunization with stabilized BG505 immunogens. One potentially confounding variable was that Abs were generated to the His-tag present in the Olio6 designs (Figure S3I). Nevertheless, additional positive correlations were found between GC Tfh cell frequencies and BG505 nAb titers (Figures S3J and S3K). The multiple correlations between GC cell populations and Ab responses suggest an overall more focused immune response toward nAbs after immunization with a stabilized trimer.

### 20 and 100 μg Env Trimer Doses Induce Comparable nAb Responses

**Figure 4:**
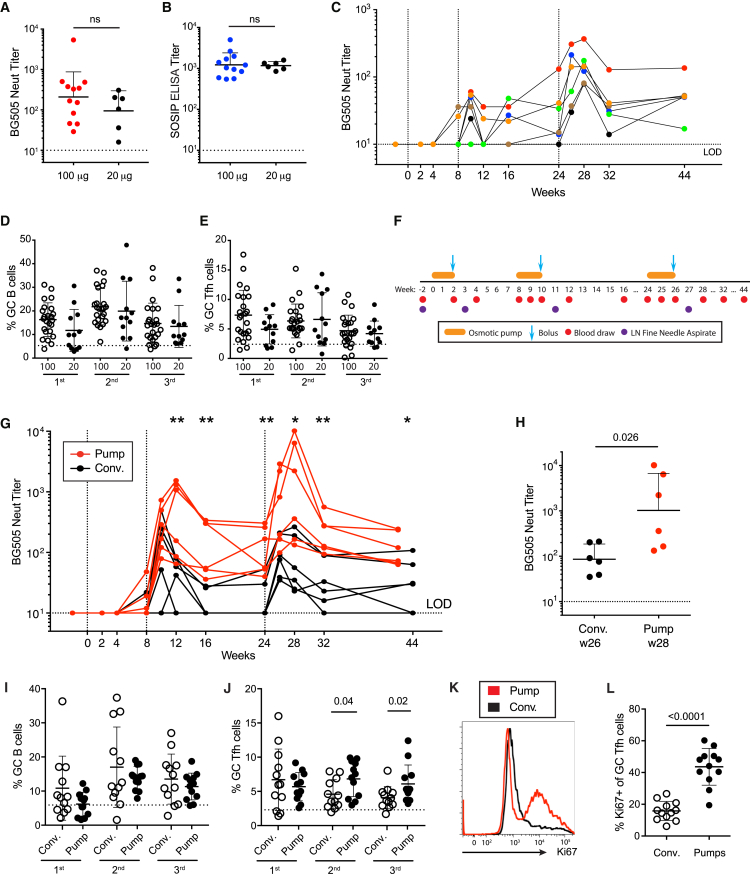
Extended Immunogen Release Induces Higher nAb Titers Than Conventional Immunization (A–E) Immunogen doses of 100 or 20 μg s.c. immunizations of BG505 SOSIP.664. (A) BG505 nAb titers at week 26 (n = 6 or 12). (B) BG505 SOSIP binding titers at week 26 (n = 6 or 12). (C) Kinetics of BG505 nAb titers. (D and E) GC B cell (D) and GC Tfh cell (E) frequencies after the first, second, and third immunizations. (F–L) Bolus (conventional) versus continuous immunogen delivery of BG505 SOSIP.v5.2 immunogen. (F) Immunization schedule and sampling for continuous antigen delivery using osmotic pumps. (G) BG505 nAb titers in animals immunized by osmotic pump (red) or conventional bolus (Conv, black) (^∗^p < 0.05; ^∗∗^p < 0.01; n = 6). (H) Peak BG505 nAb titers after the third immunization (n = 6). (I and J) GC B cell (I) and GC Tfh cell (J) frequencies after the first, second, and third immunizations. (K) Proliferation of GC Tfh cells at week 11. Flow cytometry was gated on CXCR5^hi^ PD-1^hi^ GC Tfh cells. (L) Frequency of Ki67^+^ GC Tfh cells at week 11 (n = 12). All nAb titer and ELISA binding Ab data represent geometric mean titers with geometric SD. All cell-frequency data represent the mean and SD. See also Figure S4.

To investigate the effects of immunogen dosage on nAb titer responses, we immunized six RMs with 20 μg of BG505 SOSIP.664 according to the same schedule used for the group receiving 100 μg BG505 SOSIP.664 (Figure 1A). Average GMT nAb titers 2 weeks after the third immunization were not statistically different between the 20 and 100 μg groups (Figure 4A). Additional Ab measurements at the same time point also showed no differences (Figures 4B and S4A). There was a suggestion that the kinetics of the nAb responses in the 20 μg group were distinct (Figure 4C). Thus, serum nAb titers appeared to develop somewhat more slowly in the 20 μg group, although this did not achieve statistical significance (week 10 GMT = 1:33 for 20 μg group versus 1:113 for 100 μg group, p = 0.08; Figure 4C), and nAb titers peaked at 4 instead of 2 weeks past the third immunization (peak nAb GMT for 20 μg group = 1:139 at week 28 versus 1:92 GMT at week 26; Figure 4C). GC B cell and Tfh cell frequencies were not significantly different between the 20 and 100 μg groups (Figures 4D and 4E), although there was an indication of somewhat lower frequencies after the first immunization. In conclusion, lowering the Env trimer dose from 100 to 20 μg could have altered the kinetics of the serum Ab response somewhat, but it had little effect on the development of peak tier 2 nAb.

### An Extended Immunogen-Release Strategy Induces Higher nAb Titers Than Conventional Immunization

Conventional immunizations are typically single-bolus events that deliver a volume of immunogen and adjuvant via a syringe needle. Extended immunogen release is an alternative immunization approach that could be advantageous for several reasons. First, GCs are relatively long-lasting and antigen-dependent engines of affinity maturation. As such, extended immunogen and adjuvant release could improve development of nAbs by making more trimer immunogen available during the GC response. Second, immunogens are subject to proteolysis or other protein-degradation pathways over time. Non-mechanical osmotic pumps can provide extended immunogen-release kinetics over weeks while protecting the native trimer structure until the time of release (Hu et al., 2015). Third, extended antigen availability, preferably with increased dose kinetics, mimics conditions found in natural infection and is therefore the scenario for which GCs are naturally optimized. Encouraging data in support of these ideas were obtained in mice (Hu et al., 2015, Tam et al., 2016).

To determine whether sustained immunogen delivery could enhance the nAb response in RMs, we loaded 14 day linear-release osmotic pumps with 50 μg BG505 SOSIP.v5.2 and adjuvant and implanted them subcutaneously in the left and right legs at weeks 0, 8, and 24. At the end of each immunogen delivery, RMs were each given bolus immunizations totaling 100 μg SOSIP.v5.2 to mimic dose-escalation kinetics (Figure 4F). Thus, the total dose of BG505 SOSIP.v5.2 given was 200 μg in each immunization period, whereas 100 μg was used in the control group. After a single dosing regimen, three of six pump-immunized animals had detectable nAb titers (week 8; Figure 4G). Pump delivery resulted in significantly higher nAb titers than conventional immunization, as well as shifted kinetics, after both the second and third immunizations (Figure 4G). Peak nAb titers were significantly higher in pump-immunized animals (1:1,023 GMT versus 1:86 GMT; Figure 4H). Three of six pump-immunized RMs developed nAb titers of >1:1,000. BG505 and V3-loop Ab binding titers were similarly shifted (Figures S4B–S4D). Neutralizing Ab titers of pump-immunized animals remained significantly higher than those of conventionally immunized RMs 4 months after the third immunization (Figure 4G).

In terms of LN GC reactions, B cell frequencies were similar in both pump and conventional immunization groups 3 weeks after immunization (Figure 4I), but Tfh cell frequencies were greater in pump-immunized animals after the second and third immunizations (Figure 4J). Interestingly, pump-immunized animals had significantly higher frequencies of proliferating Ki67^+^ GC Tfh cells than conventionally immunized RMs (Figure 4L). GC Tfh cells are generally minimally proliferative once a GC is established (Crotty, 2014), and therefore, the observation of robust GC Tfh cell proliferation in pump-immunized RMs is consistent with the finding that extended immunogen release changes GC characteristics and kinetics. In conclusion, slow-release immunization accelerated nAb development and enhanced peak nAb titers, and these improvements were associated with GC Tfh cell changes.

### Liposomal Presentation of Env Trimer Immunogen Generates nAbs

Liposomal presentation of native-like Env trimers as immunogens has potential advantages over soluble protein immunization. Naive B cells that recognize nAb epitopes on the HIV Env trimer are likely to be of very low affinity, and arrayed trimers on a liposomal surface can provide increased avidity for activation (Ingale et al., 2016, Steichen et al., 2016). In addition, non-neutralizing epitopes at the base of the trimer can be highly immunogenic and can inhibit development of responses to nAb epitopes (Havenar-Daughton et al., 2017, Hu et al., 2015). Liposomal presentation of immunogen could block access to these non-neutralizing epitopes.

**Figure 5:**
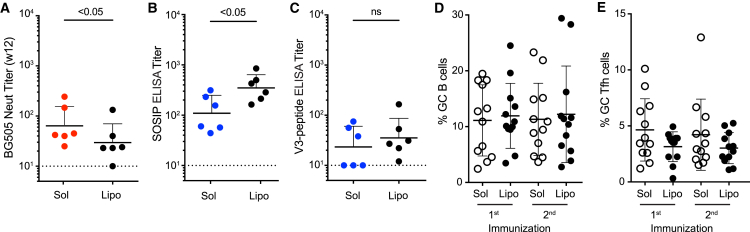
Liposomal Presentation of Env Trimer Immunogen Induces nAb Titers Comparable to Those of Soluble Immunogens RMs were immunized with soluble (Sol) BG505 Olio6 CD4-KO or BG505 Olio6 CD4-KO covalently conjugated to liposomes (Lipo). (A) BG505 nAb titers in RMs 4 weeks after the second immunization (n = 6). (B and C) BG505 SOSIP (B) or V3-loop peptide (C) binding IgG titers 2 weeks after the second immunization (week 10; n = 6). (D and E) GC B cell (D) and GC Tfh cell (E) frequencies after the first or second immunization (n = 12). All nAb titer and ELISA binding Ab data represent geometric mean titers with geometric SD. All cell-frequency data represent the mean and SD. See also Figure S5.

To investigate the effects of liposomal presentation of immunogen, we immunized six RMs with 100 μg each of BG505 Olio6 CD4-KO (day 0) and BG505 MD39 CD4-KO (week 8) covalently conjugated to liposomes. A covalent linkage of the trimer to the liposome was necessary because a non-covalent His-tag/nickel-conjugation approach was not stable for in vitro incubation in RM serum (Figures S5A and S5B). Six RMs were immunized with His-tag/nickel-conjugated BG505 SOSIP.664 (Figure S5C), but given the rapid dissociation of His-tag/nickel-conjugated liposomal particles, they were not analyzed further. In the covalently conjugated BG505 Olio6 CD4-KO liposome group, five of six animals had nAb titers at week 12 (Figure 5A). BG505 nAb titers were similar but slightly lower in the liposomal group than in the soluble immunogen group (Figures 5A and S5D). Tier 1 nAb titers were not different (Figures S5E and S5F). BG505 SOSIP binding titers were elevated in liposome-immunized RMs (Figure 5B), whereas V3-peptide IgG titers were similar (Figure 5C). GC B cell (Figure 5D) and GC Tfh cell (Figure 5E) frequencies were similar after both the first and second immunizations. Liposome immunization induced 4-fold more BG505-specific IgG^+^ blood plasmablasts (Figure S5G). Overall, the BG505 trimers covalently linked to liposomes, as used here, induced immune responses comparable to those of soluble immunogen after two immunizations.

### GC Characteristics after Each Immunization

**Figure 6:**
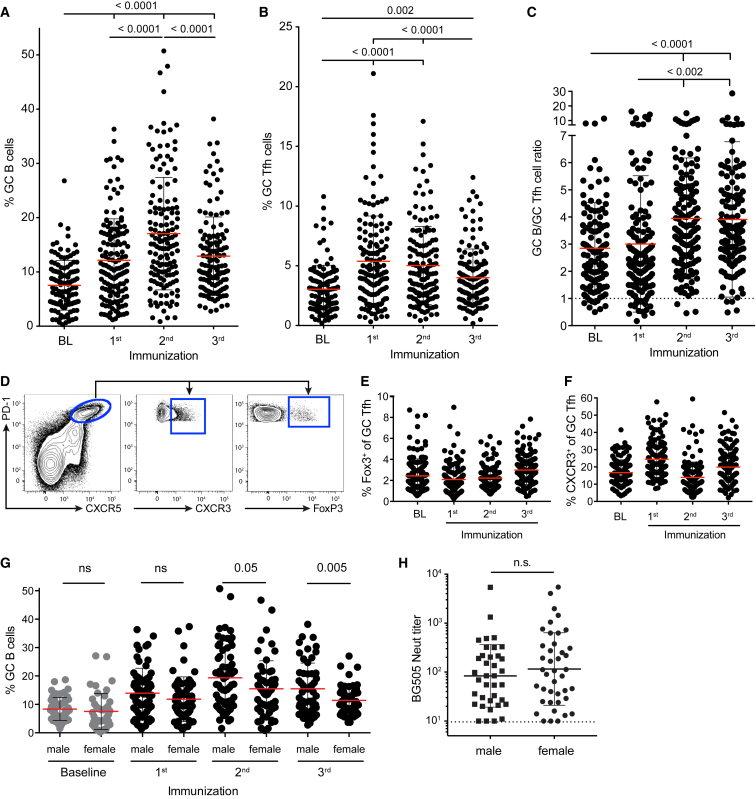
GC Dynamics across All Immunized Animals (A–C) GC B cell frequency (A), GC Tfh cell frequency (B), and ratio of GC B cells to GC Tfh cells (C) for all study animals at baseline (BL) and after the first, second, and third immunizations. Points represent individual LNs (n = 144; 72 animals × 2 LNs). (D–F) Subpopulations of GC Tfh cells. (D) Flow cytometry of GC Tfh cell sub-populations. Gates were set on the basis of total CD4^+^ T cells (Figure S6B). (E) Frequency of FoxP3^+^ GC Tfr cells. (F) Frequency of CXCR3^+^ GC Tfh cells. (G) GC B cell frequencies separated by gender. (H) BG505 nAb titers separated by gender. All nAb titer data represent geometric mean titers with geometric SD. All cell-frequency data represent the mean and SD. See also Figure S6.

Longitudinal GC responses were measured with LN FNAs for all study animals. GC B cell frequencies were significantly increased after a single Env trimer immunization when all groups were considered (p < 0.0001; Figure 6A and Table S2). The second immunization generated larger GC B cell responses than the first immunization (p < 0.0001; Figure 6A), supporting the rationale for shifting the timing of the second immunization to allow for a period of quiescence. GC Tfh cell frequencies were significantly increased after the first and second immunizations (p < 0.002; Figure 6B). GC Tfh cell and GC B cell frequencies were correlated (r = 0.67, p < 0.0001; Figure S6A). GC B cells depend on GC Tfh cells for their survival and proliferation; as such, the ratio of GC B cells to GC Tfh cells can be an indicator of the functional capacity of GC Tfh cell help (Gonzalez-Figueroa et al., 2017, Havenar-Daughton et al., 2016). The ratio of GC B cells to GC Tfh cells was significantly higher after booster immunizations, suggesting an increased Tfh helper capacity (p < 0.0001; Figure 6C). Subsets of GC Tfh cells were identified (Figures 6D and S6B); these included FoxP3^+^CXCR5^+^ T follicular regulatory (Tfr) cells and CXCR3^+^ GC Tfh cells, the latter of which are Th1 biased and could have a reduced functional helper capacity (Locci et al., 2013, Ueno et al., 2015). Both CXCR3^+^ and FoxP3^+^ GC Tfh cells were detected as minor populations among total GC Tfh cells in Env-trimer-immunized RMs (Figures 6E and 6F), but neither correlated with nAb titer (Figures S6C and S6D). In contrast, nAb titer correlated with total GC B cell frequency at multiple time points (Figure S6E). Overall, the correlations between nAb titers and GC B cells and GC Tfh cells support a central role for GC biology in the generation of HIV nAbs by immunization.

### No Gender Differences in nAb Generation

Human females have a higher propensity for developing autoimmunity and also generate higher Ab titers to immunization than males (Klein and Flanagan, 2016). The large cohort of immunized RMs permitted an examination of age, weight, and gender effects on immunization responses (Figures S6F–S6K; all data are listed in Table S1). GC B cell frequencies were higher in male than in female RMs after the second and third immunizations (Figure 6G). Gender differences in GC B cell frequency were more apparent when only the BG505-SOSIP.664-immunized RMs were considered (n = 30; Figure S6K). However, there was no statistically significant difference in BG505 nAb titers between male and female RMs (Figure 6H).

### Env-Trimer-Immunized Animals Recognize a Diversity of Autologous Neutralization Epitopes

**Figure 7:**
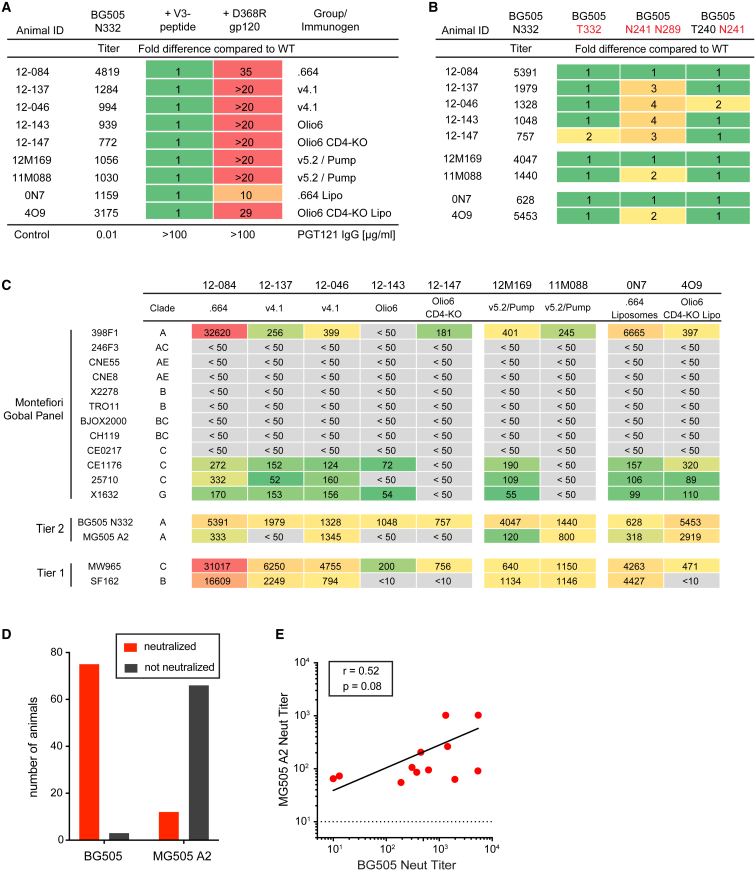
NHPs Immunized with Env Trimer Recognize Diverse Neutralization Epitopes and Generate Some Neutralization Breadth (A and B) BG505 nAb epitope mapping of sera from high-BG505-neutralizer RMs. (A) Fold difference of nAb titer in the presence and absence of competitor proteins (linear BG505 V3-peptide or BG505 D368R gp120) in sera at week 26. (B) Fold difference in nAb titers between mutant and WT BG505 pseudoviruses at week 26. (C) Neutralization breadth on a 12-virus global panel. (D) Neutralization of BG505 and MG505 A2 nAb titers at week 26 (n = 78). (E) Correlation of BG505 and MG505 A2 nAb titers at week 26 (n = 12). See also Figure S7.

Induction of autologous tier 2 nAbs in NHPs by immunization has been a high bar, and there have been very limited successes over the past three decades. Nevertheless, the ultimate goal of HIV vaccine efforts is the induction of broad nAb responses at sufficient titers to confer protection against globally diverse HIV strains. To rationally steer the immune response toward broadly neutralizing epitopes, it is necessary to understand the epitopes targeted by nAbs after Env trimer immunization. To map the epitopes targeted by the autologous tier 2 nAbs, we selected the nine RMs displaying the highest BG505 neutralization titers, ranging from 1:628 to 1:5,453. D368R-mutated gp120 fully absorbed the tier 2 neutralizing responses in all tested animals, indicating that the targeted epitopes are on monomeric gp120 and are unlikely to be the CD4 binding site, for which D368 binding interactions are generally essential (Figures 7A and S7A). Linear BG505 V3 peptides did not compete with nAb activity from any of the sera (Figures 7A and S7A). To refine targeted epitopes within gp120, we evaluated sera for neutralization of pseudovirus glycan variants. BG505 variant S241N P291S N332 introduces glycans at positions N241 and N289 to fill in the “glycan hole” that is targeted in about two-thirds of BG505-SOSIP-immunized rabbits (McCoy et al., 2016). Four of nine RMs displayed a >2-fold titer decrease on BG505 S241N P291S N332 virus (Figures 7B and S7B), indicating that the hole is targeted in some RMs. When tested on the BG505 P240T S241N N332 virus, which only partially fills in the glycan hole and corrects for an unusual angling of the N241 glycan, only one of nine animals displayed a 2-fold reduction in nAb titer, suggesting that the majority of hole-directed nAbs center on the N289 site (Figure 7B). However, even for the animals that did target the hole, the response was less dominant for RMs than for rabbits, and other neutralizing responses were most likely present. Finally, sera were tested against BG505 T332 pseudovirus, which lacks the glycan at position N332, the central glycan of a frequently targeted broadly neutralizing epitope cluster (Landais et al., 2016, Walker et al., 2010). None of the RMs showed evidence of targeting this epitope cluster (Figure 7B). In sum, the top neutralizer RMs recognized diverse nAb epitopes on gp120, including but not limited to the N241-N289 region.

### Induction of Neutralization Breadth after Env Trimer Immunization

We used a commonly used panel of 12 heterologous tier 2 viruses representative of global HIV strain diversity (deCamp et al., 2014) to assess neutralization breadth. Remarkably, the majority of top neutralizer animals tested were able to neutralize three to four viruses of the 12-virus panel at moderate potency (Figure 7C). The MG505 A2 virus diverges from BG505 by only 17 amino acids. Thus, we used MG505 A2 as an alternative approach to measure breadth. Although almost all animals in this study developed nAbs to BG505 pseudovirus, only 15% of animals neutralized MG505 A2 (n = 12/78; Figure 7D). Among animals that neutralized MG505 A2, peak BG505 nAb GMTs were significantly above average (1:460 versus 1:99 GMT; Figure S7C), suggesting that animals with higher autologous neutralization responses had a higher likelihood of developing neutralization breadth. BG505 and MG505 A2 neutralization titers in those animals roughly correlated (Figure 7E). The tier 2 neutralizing epitopes on MG505 A2 could be mapped to the V1-loop for three of the nine top neutralizer RMs, which could be indicative of the BG505 neutralizing epitopes (Figures S7D–S7F). Overall, some neutralization breadth was observed for animals immunized with BG505 trimer, particularly among those with high BG505 autologous nAb titers.

## Discussion

The SOSIP molecule, with its close mimicry of the native HIV envelope spike, is a promising component of an HIV vaccine to induce protective Abs (Sanders and Moore, 2017). However, a concern has been the rather inconsistent induction of nAbs by SOSIP immunization in NHPs, particularly given that NHPs are often seen as a good model for predicting human responses to vaccination. Here, we now show reliable induction of nAbs after BG505 SOSIP.664 immunization.

Four features of the immunization protocol could have contributed to this success: (1) a longer time interval between immunizations, (2) a strong adjuvant, (3) bilateral immunizations, and (4) s.c. administration. First, a 0-8-24 week immunization schedule was used. The longer interval between immunizations could have allowed the GC response to more fully mature after each immunization, thus generating the somatic hypermutation (SHM) needed for gaining B cell receptor affinity for neutralizing epitopes. Indeed, we demonstrated that an 8 week boost was more effective than a 6 week boost at inducing GC B cells and nAbs. Second, the strong GC-inducing adjuvant ISCOMATRIX was used. ISCOMATRIX had been used in some, but not all, previous studies of SOSIP trimer immunization in RMs. Third, bilateral immunizations were used to increase the number of draining LNs and putatively engage twice as many antigen-specific CD4 T cells and B cells specific to neutralizing epitopes. Fourth, s.c. immunization was directly compared with i.m. immunization and proved to be significantly superior in terms of both the rate of development and the magnitude of the induced nAb response. s.c. immunizations permit more efficient drainage to surrounding LNs than i.m. immunization. An increase in intact native-like Env trimer reaching GCs within draining LNs most likely encourages SHM and favors B cells with nAb specificities over those with non-neutralizing specificities, such as those targeting Env-breakdown products. Bilateral s.c. immunizations of BG505 SOSIP.664 given with a strong adjuvant at lengthier boosting intervals induced strong nAb responses in the majority of RMs.

Also, in contrast to earlier studies, our study generated substantial tier 2 nAb titers by week 10 after only two immunizations. The immunization parameters outlined above—adjuvant, timing, and bilateral s.c. injection—most likely contributed to the rapid nAb generation, which was more rapid than the typical development of autologous nAbs after 3–6 months in HIV-infected individuals (Richman et al., 2003, Wei et al., 2003). It is encouraging that autologous nAbs can potentially be developed faster after protein immunization than HIV infection, suggesting the possibility of developing broadly neutralizing Abs via serial immunizations faster than they develop in HIV-infected people.

To interrogate the immunological underpinnings of successful nAb generation, we acquired LN FNAs throughout the study, allowing us to examine GCs within the draining LNs. GC B cell frequencies measured at just 3 weeks after the priming immunization predicted the development of nAb generation after further immunizations. Moreover, the GC B cell frequency tracked with nAb titer after each immunization, emphasizing the pivotal role of GCs in the generation of nAb responses. Optimally engaging GC responses through the choice of adjuvant, immunization schedule, and antigen delivery is a means of positively affecting nAb generation and should bear careful consideration. Of note, the third immunization accomplished relatively little beyond moderately increasing the nAb titers of RMs with low titers after the second immunization.

Among all immunization groups, we obtained the highest nAb titers by extending immunogen release. This approach appeared to outperform the conventional immunization strategy with the same immunogen. A 2 week osmotic pump coupled with a bolus injection of immunogen to mimic a dose-escalating immunization significantly increased tier 2 nAb titers, favorably shifted Ab-response kinetics, and enhanced GC activity. One caveat is that the extended-immunogen-release group received a total of 200 μg immunogen per immunization, whereas 100 μg was used in the control group. It is therefore possible that the larger total antigen dose could have been beneficial for nAb development. However, we observed only marginal differences between 20 and 100 μg immunizations, supporting the interpretation that the increased titers in the extended-immunogen-release group were due to the immunization strategy. Future studies with dose-matched groups are needed. Nevertheless, the data show that nAb development was dramatically modulated in the extended-immunogen-release group.

Liposome presentation is an appealing means of presenting Env trimers to minimize exposure of non-neutralizing epitopes. Disappointingly, we found no significant improvements in tier 2 nAb titers between liposome-displayed and soluble Env trimers. This could have arisen for several potential reasons, including the stability of the liposome preparations in vivo, which will require further exploration before any definitive conclusions can be drawn about the utility of liposome-displayed trimers.

Previous studies have indicated that tier 1 non-nAb epitopes might interfere with induction of tier-2-epitope-directed nAb responses, given that naive B cells with tier 2 specificities can be rare or have low affinity and be disadvantaged in the competition for antigen and T cell help (Havenar-Daughton et al., 2017, McGuire et al., 2014). Consequently, efforts have been made to design soluble native Env trimers with improved antigenic properties, whereby epitopes such as the V3-loop tip and the CD4-induced epitopes are constrained in conformations that preclude Ab recognition. We tested several of the latest-generation stabilized BG505 trimer designs head-to-head. The BG505 Olio6 designs showed the strongest reduction in tier 1 nAb titers. However, limiting the V3-loop tip Ab response did not redirect the immune response sufficiently to improve tier 2 nAb titers. This could be because other specificities, such as epitopes on the exposed base of the trimer, still precede the neutralizing epitope specificities in the immunodominance hierarchy (Havenar-Daughton et al., 2017).

Finally, the autologous tier 2 nAb responses generated by BG505 Env trimer immunogens in this study are an important benchmark for the HIV vaccine field. Cross-reactive tier 2 nAb breadth is the next important goal. In using a single BG505 strain for immunization, we did not expect to generate a broad nAb response, and in the majority of RMs, indeed we found no evidence of MG505 cross-reactive tier 2 nAbs. Some nAb breadth was detected on a 12-virus global panel in RMs with the highest BG505 nAb titers. RMs immunized with BG505 Env trimer recognized a larger diversity of neutralizing epitopes than did rabbits, which is encouraging for future development of nAb breadth. In addition, the new immunization regimen rapidly and reproducibly induced autologous nAbs, which will be a boon to future immunization studies aimed at generating nAb breadth because it will allow for faster testing and iteration of novel immunogen designs.

## STAR★Methods

### Key Resources Table

**Table:** 

REAGENT or RESOURCE	SOURCE	IDENTIFIER
**Antibodies**		

mouse anti-Ki67 Alexa Fluor 488 (Clone B56)	BD Biosciences	Cat# 558616; RRID: AB_647087
mouse anti-PD-1 PE (Clone EH12.2H7)	BioLegend	Cat# 329905; RRID: AB_940481
mouse anti-Bcl6 PE-CF594 (Clone K112-91)	BD Biosciences	Cat# 562401; RRID: AB_11152084
mouse anti-ICOS PerCP-cy5.5 (Clone C398.4A)	BioLegend	Cat# 313517; RRID: AB_10639735
mouse anti-CXCR5 PE-Cy7 (Clone MU5UBEE)	Thermo Fisher Scientific	Cat# 25-9185-42; RRID: AB_2573540
mouse anti-FoxP3 V450 (Clone 259D/C7)	BD Biosciences	Cat# 561182; RRID: AB_10564088
mouse anti-CD4 BV650 (Clone L200)	BD Biosciences	Cat# 563737
mouse anti-CD8 BV786 (Clone RPA-T8)	BD Biosciences	Cat# 563823
mouse anti-CXCR3 BUV395 (Clone 1C6/CXCR3)	BD Biosciences	Cat# 565223
mouse anti-CD20 BUV737 (Clone 2H7)	BD Biosciences	Cat# 564432
mouse anti-CD95 APC (Clone DX2)	BD Biosciences	Cat# 561978; RRID: AB_10895117
mouse anti-CD3 Alexa Fluor 700 (Clone SP34.2)	BD Biosciences	Cat# 557705; RRID: AB_396814
mouse anti-CD27 APC-e780 (Clone O323)	Thermo Fisher Scientific	Cat# 47-0279-42; RRID: AB_1272040
Fixable Viability Dye Aqua	Invitrogen	Cat# L34966

**Bacterial and Virus Strains**		

BG505.W6M.ENV.C2	Wu et al., 2006 / NIH AIDS Reagent Program	Cat# 11518
MG505.W0M.ENV.A2	Wu et al., 2006 / NIH AIDS Reagent Program	Cat# 11528
MG505.W0M.ENV.H3	Wu et al., 2006 / NIH AIDS Reagent Program	Cat# 11529
12 virus panel – global isolates	deCamp et al., 2014	N/A

**Chemicals, Peptides, and Recombinant Proteins**		

ISCOMATRIX	CSL Behring	N/A
BG505 SOSIP.664 (HEK293T produced)	Sanders et al., 2013	N/A
BG505 SOSIP.664 (CHO produced)	Catalent	N/A
BG505 SOSIP.v4.1	de Taeye et al., 2015	N/A
BG505 SOSIP.v5.2	A.T.d.l.P. et al., unpublished data	N/A
BG505 NFL	Sharma et al., 2015	N/A
BG505 Olio6	D.W.K. et al., unpublished data	N/A
BG505 Olio6 CD4-KO	D.W.K. et al., unpublished data	N/A
BG505 V3-peptide TRPNNNTRKSIRIGPGQAFYATG	A&A Labs LLC	N/A
BG505 V3-peptide biotin TRPNNNTRKSIRIGPGQAFYATGGAGK-biotin	GenScript	N/A
TAPNNFTVKSIRIGPGAFYYMGGAGK-biotin	GenScript	N/A
His-Tag biotin peptide biotin-ALDGGGGSHHHHHHHH	A&A Labs LLC	N/A
Evans Blue dye	Sigma Aldrich	Cat# E2129
DMPC: 1,2-dimyristoyl-sn-glycero-3-phosphocholine	Avanti Polar Lipids	Cat# 850345
DGS-NTA(Ni): 1,2-dioleoyl-sn-glycero-3-[(N-(5-amino-1-carboxypentyl) iminodiacetic acid)succinyl] (nickel salt)	Avanti Polar Lipids	Cat# 790404
MPB: 1,2-dioleoyl-sn-glycero-3-phosphoethanolamine-N-[4-(p-maleimidophenyl)butyramide] (sodium salt)	Avanti Polar Lipids	Cat# 870012
Cholesterol	Sigma-Aldrich	Cat# C8667

**Experimental Models: Cell Lines**		

TZM-bl cells	NIH AIDS Reagent Program	Cat# 8129

**Experimental Models: Organisms/Strains**		

Indian-origin rhesus macaques (outbred)	AlphaGenesis Inc	N/A

**Recombinant DNA**		

pSG3Δenv plasmid	NIH AIDS Reagent Program	Cat# 11051

**Software and Algorithms**		

Prism v7.0	GraphPad	https://www.graphpad.com/scientific-software/prism/
FlowJo v9.9.4	FlowJo	https://www.flowjo.com

**Other**		

Mini-osmotic pumps (0.5 μL/hr, 14 day release)	ALZET	Model 2002

### Contact for Reagent and Resource Sharing

Further information and requests for resources and reagents should be directed to and will be fulfilled by Dennis Burton (burton@scripps.edu).

### Experimental Model and Subject Details

#### Rhesus Macaques

Outbred Indian RMs *(Macaca mulatta)* were sourced and housed at Alphagenesis, Yemasee, SC and maintained in accordance with NIH guidelines. These studies were approved by the appropriate Institutional Animal Care and Use Committees (IACUC). None of the NHPs were previously enrolled in other studies, and animals were randomly allocated to experimental groups. The majority of macaques were 3–4 years of age at the time of first immunization (Figure S6F). Body weight and sex of animals are listed in Figures S6G and S6H.

### Method Details

#### Macaque Immunizations

Animals were immunized at 3 time points: week 0, week 8, and week 24. All immunizations were administered as split doses. Unless otherwise stated, each immunization consisted of two s.c. injections of 50 μg of Env trimer protein + 37.5 units (U) of ISCOMATRIX adjuvant (CSL), composed of cholesterol, phospholipid, and saponin (Sun et al., 2009), in sterile phosphate-buffered saline (PBS) diluent for a total of 100 μg of Env trimer protein + 75 U of ISCOMATRIX per immunization per animal. s.c. immunizations were given in a volume of 0.5mL with a 1 inch, 25-gauge needle at the medial inner mid-thigh of each leg. The s.c. injection technique consists of making a “skin tent” and inserting the needle into the s.c. space at a 45° angle. The i.m. immunizations were administered in the quadriceps muscle of each thigh with a 1 inch, 25-gauge needle at a 45° angle (0.5 mL/thigh). One group of animals was immunized with a lower total dose of immunogen (total, 20 μg of Env trimer protein + 75 U of ISCOMATRIX). The s.c. BG505 SOSIP.664 immunization group included 12 animals to increase statistical power in comparisons to other groups of 6 animals.

s.c. pump immunization (ALZET, Model 2002) and bolus injection were used to mimic an escalating dose immunization. Each 14-day osmotic pump filled with 50 μg of BG505.v5.2 SOSIP + 37.5 U ISCOMATRIX was implanted under the skin of each inner thigh and sutured in place to prevent potential removal by the animal. 14 days later, 50 μg BG505.v5.2 SOSIP + 37.5 U ISCOMATRIX (CSL) was given via s.c. bolus injection in each inner thigh as described above. Pumps were removed at day 18 to avoid interference with the bolus immunization.

#### Sample Collection

Serum was collected in SST Vaccutainer tubes (BD Biosciences) and processed according to the manufacturer’s instructions. Multiple aliquots of 0.5 mL were frozen at −80°C. Whole blood was collected in K2 EDTA Vaccutainer tubes (BD Biosciences) for plasma and PBMC isolation. Multiple aliquots of 0.5 mL of plasma were frozen at −80°C. PBMCs were isolated using Thermo Fisher Scientific Nunc EZFlip Conical Centrifuge Tubes per manufacturer’s instructions. PBMCs were isolated, counted, and re-suspended at 1 × 10^7^ cells/mL in FBS containing 10% DMSO. Aliquots were subsequently frozen in 1 mL vials using a Mr. Frosty freezing container (Nalgene, cooling rate of 1°C / minute) and placed in a −80°C freezer. The following day PBMC samples were moved to storage in a liquid nitrogen freezer tank.

LN FNAs were used to sample both right and left inguinal areas inferred to be the primary draining LNs, 3 weeks post each immunization (Havenar-Daughton et al., 2016). A veterinarian performed the LN FNAs on palpable superficial inguinal LNs. Forceps were used to isolate and elevate the LN. A 22-gauge, 1” needle attached to a 3 mL syringe was inserted into the LN and moved in a back and forth motion four times. No suction was applied. Cells in the needle were ejected into cold RPMI + 10% FBS media in 15 mL tubes and the needle was flushed repeatedly to collect all cells. 15 mL tubes were filled with cold media, wrapped in bubble wrap to prevent freezing, packaged with frozen cold packs, and shipped the same day for further processing and analysis at Beth Israel Deaconess Medical Center. Red blood cell lysis was generally not necessary, but was performed on a few samples using ACK lysis buffer for 3 min. All LN FNA samples were stained and fixed for flow cytometry within 18–24 hr of biopsy. Successful LN FNAs were obtained (i.e., all the way to quantifiable FACS data) in 98% (541 of 550) of cases. A median of 3 × 10^6^ cells was recovered.

#### Evans Blue Dye Extraction and Quantification

One mL (72 hr study) or 0.5 mL (48 hr study) of a 0.2% Evans Blue dye solution was delivered via s.c. or i.m. injection into the thigh of RMs, respectively. LNs were harvested after 48 hr (one animal) or 72 hr (two animals) and fixed in freshly made 4% PFA for 24 hr. Each LN was cut into small pieces and incubated in 500 μL of formamide (Sigma, Cat# F-7503) for 24 hr at 60°C to extract the dye. Fluorescence of the extracted dye in supernatant was measured (excitation at 620 nm, emission at 680 nm) using a Tecan Infinite M200 Pro plate reader and quantified using a standard curve. LNs injected with known titrated amounts of Evans Blue and processed in the same manner showed that the extraction process recovered 77 ± 5% of LN-associated dye; importantly this efficiency was independent on the amount of dye present.

#### BG505 Native-like Env Trimer Immunogens

##### Expression of BG505 SOSIP.664 Variants

BG505 SOSIP.664 (also referred to as BG505 SOSIP.v3.2 (de Taeye et al., 2015) was expressed in stable HEK293T cell lines for material used in all BG505 SOSIP.664 groups except for one i.m. immunized group, in which BG505 SOSIP.664 was stably expressed in CHO cell lines (Chung et al., 2014). We found no significant differences in immunogenicity between RM immunized i.m. with HEK293T or CHO cell-produced BG505 SOSIP.664 and subsequently combined data from the two groups for increased statistical power. BG505 SOSIP.664 from both cell lines was purified using a 2G12-affinity column followed by size exclusion chromatography (SEC). These proteins had no His-tag. Fractions corresponding to trimer were pooled and concentrated down to between 0.3-0.5 mg/mL, filter sterilized, aliquoted, and flash frozen. The aliquots were stored at −80°C until dry ice shipment to Alphagenesis Inc. BG505 SOSIP.v4.1 (de Taeye et al., 2015) and BG505 SOSIP.v5.2 (A.T.d.l.P. et al., unpublished data) were expressed in HEK293F cells by transient co-transfection with furin. BG505 SOSIP.v4.1 contains a A316W mutation, which improves hydrophobic packing and stability of the V3-loop, and a E64K mutation, which reduces spontaneous sampling of the CD4-bound “open” trimer conformation. The BG505 SOSIP.v5.2 builds upon the v4.1 design, with the addition of a second disulfide bond between gp120 and gp41 to further increase trimer stability. The proteins were purified using methods described elsewhere (Pugach et al., 2015), with either 2G12-affinity (BG505 SOSIP.v4.1) or PGT145-affinity columns (BG505 SOSIP.v5.2) followed by SEC. These proteins had no His-tag. Fractions corresponding to trimer were pooled and concentrated down to ∼2.3 mg/mL (BG505 SOSIP.v4.1) or ∼0.8 mg/mL (BG505 SOSIP.v5.2). Structural validation of trimers was performed by analyzing negative-stain electron microscopy (EM) 2D class averages following methods described previously (de Taeye et al., 2015). All samples were filter sterilized prior to aliquoting and flash freezing. Aliquots were stored at −80°C until dry ice shipment to the Alphagenesis Inc.

##### Expression of BG505 NFL Trimers

The BG505 NFL sequence was previously reported (Sharma et al., 2015) and includes a C-terminal His-tag. BG505 NFL was transiently expressed in HEK293F cells. In brief, 350 μg of BG505 NFL plasmid DNA was mixed with 1 mL of 293-fectin transfection reagent (Invitrogen), incubated for 20 min at 37°C and subsequently transfected into 1 l of 293F cells with cell density of 1.5 million/ml and 99% viability. The cells were grown for 4 days in a CO_2_ incubator shaker at 37°C. Cells were harvested at 4000 rpm at 25°C for 30 min and the culture supernatant was sterile filtrated (0.22 μm, Nalgene) to remove any remaining cell debris. The BG505 NFL trimer was purified via affinity chromatography by passing the supernatant over *Galanthus nivalis* (GLN)-lectin-agarose (Vector Laboratories) columns at 4°C. Columns were washed extensively with PBS containing 500 mM NaCl, followed by PBS containing 10 mM methyl-α-D-mannopyranoside. Bound protein was eluted with PBS containing 500 mM NaCl and 500 mM methyl-α-D-mannopyranoside. The eluted BG505 NFL protein was concentrated in Amicon Ultra-15 centrifugal filter tubes (100 kDa MWCO, Millipore) to 500 μl and subjected to SEC using a Superdex 200 10/300 GL column, to purify the trimer population. SEC eluted fractions were analyzed by native-PAGE, trimer fractions were pooled, and then further purified by negative selection using an F105-Ab affinity column. Aberrant trimers bind to F105 mAbs, while the flow through contains 100% native-like, well-ordered BG505 NFL trimers. A detailed protocol for negative selection using an F105-Ab column is described elsewhere (Sharma et al., 2015). The purified, native-like, BG505 NFL trimer immunogen was characterized by blue native-PAGE, ELISA, and Biolayer interferometry (BLI) binding analysis before vaccine formulation. Here, ELISA and BLI binding assays were used as diagnostic tools to assess the structural integrity and native-like features of BG505 NFL trimers. In both assay formats, strong binding of trimer sensitive bNAbs (PGT145, PGDM1400, VRC26, PG16, VRC06, and PGT151) and other select bNAbs (2G12, VRC01, and PGT121) was detected, while only very weak to no binding of non-neutralizing Abs (F105, b6, F240, and 7B2) was detected. Thus all BG505 NFL protein used in RM immunizations was entirely composed of well-ordered, native-like trimers.

##### Expression of BG505 Olio6 and Related Trimers

The BG505 Olio6, BG505 Olio6 CD4-KO and BG505 MD39 CD4-KO proteins were designed with computational protein design methods using the Rosetta Design platform. The trimers are mutational variants of BG505 MD39 (Steichen et al., 2016). BG505 Olio6 is based on the BG505 SOSIP MD39 design (Steichen et al., 2016) that contained sequence modifications that increase trimer stability and yield, and also reduced V3 reactivity compared to BG505 SOSIP.664. Further stabilization of the V3-loop in BG505 Olio6 was achieved through computational design of complementary mutations in the V3-loop and the underlying gp120 (non-V3 gp120 mutations not shown in Figure 3A), creating a hydrophobic cluster of residues to anchor V3. BG505 Olio6 was then modified via multi-state structural design to reduce CD4 binding affinity while retaining CD4-binding-site-directed bnAb affinity, resulting in BG505 Olio6 CD4-KO. This CD4 mutation is hypothesized to increase the effective dose of circulating immunogen in vivo and decrease the exposure of CD4-binding-induced non-neutralizing epitopes by eliminating human CD4-induced trimer opening. BG505 Olio6, BG505 Olio6 CD4-KO and BG505 MD39 CD4-KO proteins included His-tags and were purified by Ni++ affinity chromatography followed by SEC, as described previously (Steichen et al., 2016). Design and biophysical characterization of BG505 Olio6 and BG505 Olio6 CD4-KO are described elsewhere (D.W.K. et al., unpublished data).

#### Liposome Synthesis and Characterization

Trimer-conjugated liposomes were prepared as previously described with some modifications (Steichen et al., 2016). In brief, unilamellar liposomes were comprised of DMPC:cholesterol:DGS-NTA(Ni):MPB lipids in a 61.5:28.5:5:5 molar ratio. Lipids 1,2-dimyristoyl-sn-glycero-3-phosphocholine (DMPC), 1,2-dioleoyl-sn-glycero-3-[(N-(5-amino-1-carboxypentyl) iminodiacetic acid)succinyl] (nickel salt) (DGS-NTA(Ni)) and 1,2-dioleoyl-sn-glycero-3-phosphoethanolamine-N-[4-(p-maleimidophenyl)butyramide] (sodium salt) (MPB) were purchased from Avanti Polar Lipids. Cholesterol was purchased from Sigma-Aldrich. Liposomes were synthesized by lipid film rehydration and membrane extrusion, followed by post-synthesis binding of 6xHis-Cys C-terminal modified trimer for 1 hr at 37°C in PBS followed by overnight incubation at 4°C with rotation. Covalent coupling could also be performed using trimers without C-terminal cysteines for proteins containing a lysine directly adjacent to the 6× His-tags, as maleimides also react with primary amines in the absence of cysteine; coupling in this manner showed similar trimer retention in serum. Non-covalent liposomes were prepared similarly without MPB lipid, with DMPC:cholesterol:DGS-NTA(Ni) lipids in a 66.5:28.5:5 mole ratio. Unconjugated trimer was removed by size exclusion chromatography using a Sepharose CL-2B resin (Sigma). Total conjugated trimer was quantified by ELISA in the presence of 1% triton-X and 100 mM imidazole to fully disrupt liposomes and Ni-6xHis interactions, respectively. Trimer was captured on Nunc MaxiSorp plates coated with (mouse Fc) VRCO1 and detected using PGT151, followed by secondary detection with a goat anti-human IgG-HRP conjugate. Antigenic profiles were similarly determined by ELISA on intact liposomes captured on VRC01-coated plates. To ensure batch-to-batch homogeneity, trimer-conjugated liposomes were also characterized by dynamic light scattering and cryo-electron microscopy (Jeol 2100F TEM) in the Swanson Biotechnology Center at the Koch Institute, MIT.

For stability analysis, trimer-conjugated liposomes were incubated with 20% (final concentration) rhesus serum or PBS (control) for up to 14 days after which released trimer was separated from trimer-conjugated liposomes by size exclusion chromatography as described above. Intact trimer in each elution fraction was quantified exactly as described above using PGT151 as the detection Ab. Serum stability was determined to be equivalent (data not shown) for covalent liposomes functionalized with trimers either with or without C-terminal cysteines (but with a neighboring lysine).

#### Pseudovirus Neutralization Assays

Replication incompetent HIV pseudovirus was produced by co-transfecting *env* plasmids with an *env*-deficient backbone plasmid (pSG3Δ*env*) in HEK293T cells in a 1:2 ratio, using the X-tremeGENE 9 transfection reagent (Roche). Pseudovirus was harvested after 48–72 hr by sterile-filtration (0.22 μm) of cell culture supernatants, and neutralization was tested by incubating pseudovirus and serum or mAbs for 1 hr at 37°C before transferring them onto TZM-bl cells as previously described (Sok et al., 2016). For competition neutralization assays, BG505 D368R gp120 or BG505 V3-peptide (TRPNNNTRKSIRIGPGQAFYATG) was added at a concentration of 25μg/ml. Similar assays were performed at Duke University (Li et al., 2005). Neutralization is measured in duplicate wells within each experiment. BG505 nAb titers for group comparisons were measured in three or more independent experiments that were subsequently averaged. The BG505 time course neutralization data shown in Figures 1C, 4C, and 4G were generated in three large experiments, respectively, to test sera from all time points side-by-side, thus ensuring the highest nAb titer comparability between time points. Of note, all neutralization assays used serum samples, as plasma samples, depending on the anti-coagulant used, can interfere with TZM-bl neutralization assays. Neutralization was tested starting at 1:10 serum dilutions followed by nine serial 3-fold dilutions to ensure highest sensitivity and range of detection. Neutralization IC_50_ titers were calculated using the “One site – Fit logIC_50_” regression in Graphpad Prism v7.0. IC_50_ nAb titers of incomplete neutralization curves that reached at least 50%, but less than 90% maximal neutralization, were calculated by constraining the regression fit through 0% and 100% neutralization, to ensure accurate calculation of half-way (50%) nAb titers. All neutralization titers are reported as IC_50_ titers. All nAb titer data panels show geometric mean titers with geometric SD.

BG505 pseudovirus neutralization was tested using the BG505.W6M.ENV.C2 isolate (Wu et al., 2006), carrying the T332N mutation to restore the N332 glycosylation site, unless stated otherwise. The related maternal MG505 A2 and H3 isolates (MG505.W0M.ENV.A2 and MG505.W0M.ENV.A2, respectively) only differ in 2%–3% in amino acid residues (17 and 30 aa changes, respectively) compared to BG505, and were used to gauge neutralization breadth and to map serum nAb epitopes (Wu et al., 2006). All indicated mutant pseudoviruses were created by site-directed mutagenesis of the respective *env* plasmid and verified by Sanger sequencing. All mutant pseudoviruses were checked for intact antigenicity of epitopes not affected by the respective mutations (see Figure S7B). Heterologous neutralization breadth was tested on a panel of 12 cross-clade isolates, reported to be most representative of larger virus panels composed of global isolates from diverse clades (deCamp et al., 2014).

#### SOSIP.664 Serum IgG ELISA Binding Assays

Microlon 96-well plates (Corning) were coated overnight with streptavidin at 2.5 μg/mL (Thermo Fisher Scientific) or mouse anti-His Ab at 2.5 μg/mL (Thermo Fisher Scientific) at 2.5 μg/mL in phosphate-buffered saline (PBS) at 50 μL per well. Plates were then washed four to five times with PBS-tween (0.05%) and blocked with PBS + 3% BSA for 1 hr at room temperature. If capturing biotinylated BG505 SOSIP.664-Avi gp140, BG505 NFL-His gp140, or BG505-Avi gp120, proteins were added at 1 μg/mL in PBS + 1% BSA for 2 hr at room temperature. Plates were then washed four to five times with PBS-tween (0.05%) and serially diluted sera in PBS + 1% BSA were then added for 1 hr at room temperature. Plates were then washed four to five times with PBS-tween (0.05%) and alkaline phosphatase-conjugated goat anti-human IgG (Jackson ImmunoResearch) was added for 1 hr at a 1:1,000 dilution (final concentration 0.33 μg/mL) in PBS + 1% BSA at room temperature. Plates were then washed four to five times with PBS-tween (0.05%) and absorption at 405 nm was measured following addition of phosphatase substrate in alkaline phosphatase buffer. We calculated half maximal EC_50_ binding titers using GraphPad Prism v7.0. All ELISA Ab data panels show geometric mean titers with geometric SD.

#### Peptide IgG ELISA Binding Assays

V3-peptdie ELISA assays were performed exactly as SOSIP.664 ELISAs, with the following modifications. For the BG505 Olio6 versus WT cross-binding comparison (Figure 3D) and to assess V3-peptide antigenicity (Figure S3D), Microlon 96-well plates (Corning) were coated overnight with streptavidin at 2.5 μg/mL (Thermo Fisher Scientific) in phosphate-buffered saline (PBS). After blocking, C-terminally biotinylated BG505 V3-peptides (Olio6 TAPNNFTVKSIRIGPGQ-AFYYMGGAGK-biotin or SOSIP.664 TRPNNNTRKSIRIGPGQAFYATGGAGK-biotin) were added at 2.5 μg/mL in PBS + 1% BSA for 2 hr at room temperature. All other V3-peptide binding assays were done by directly coating BG505 V3-peptide (TRPNNNTRKSIRIGPGQAFYATG) to Microlon 96-well plates at 2.5 μg/mL in PBS overnight, with comparable results to biotin/streptavidin capture (data not shown). His-tag peptide ELISA assays were performed as the SOSIP.664 ELISAs above, with the following modification: a N-terminally biotinylated His-tag peptide (biotin-ALDGGGGSHHHHHHHH) was coated as the antigen (2.5μg/ml in PBS + 1% BSA). All ELISA Ab data panels show geometric mean titers with geometric SD.

#### Flow Cytometry

Fresh cells from LN FNA samples were washed, counted, and stained with a panel of antibodies (see key resource table) to identify GC Tfh cells and GC B cells. Each animal was immunized in both the right and left legs, yielding two (right and left) draining inguinal LN samples per animal. Samples were acquired with a LSR-II flow cytometer (BD Biosciences). FlowJo 9.9 (Tree Star) was used for analysis. Example gating schemes are shown in Figure S1E. All cell frequency data panels show mean frequency and SD.

#### Plasmablast ELISPOT

BG505-specific Ab-secreting cells (ASCs) were detected by Enzyme-Linked ImmunoSpot (ELISPOT) assay on fresh cells from blood, obtained 5 days after the second immunization. 96-well multi-screen HTS HA filter plates, opaque (Millipore) were coated with 20 μg/mL of unconjugated *Galanthus nivalis* lectin (Vector labs) to capture antigen. Plates were coated overnight, washed, and blocked with complete RPMI medium at 37°C. 20 μg/mL of BG505 SOSIP.664 protein (laboratory of J.P. Moore, Cornell University) was added and incubated 1 hr at 37°C. All washes for GNL-BG505 plates were performed with PBS only. Plates were washed, mononuclear cells were plated in 3-fold serial dilutions, and incubated overnight 37°C. Plates were washed and incubated for 2 hr at RT with anti-monkey biotin conjugated IgG (Rockland) diluted at 1/1,000 in PBS with 1% FBS solution. Plates were washed and HRP avidin D (Vector labs) was added at 1/1,000 dilution and incubated for 2 hr at RT. The last wash was performed with PBS-T followed by PBS. An AEC Substrate Set (BD Biosciences) was used to develop spots. Plates were scanned and counted at Zellnet Consulting using the Immunospot CTL image acquisition (Cellular Technologies). Spots are reported as antigen-specific ASCs per million mononuclear cells.

### Quantification and Statistical Analysis

Graphpad Prism v7.0 was used for all statistical analyses. The significance of differences in neutralization and binding data between groups was calculated using unpaired, two-tailed Mann-Whitney U tests. Correlations between neutralization and binding datasets were calculated using log transformed Ab titer values in two-tailed Pearson correlation tests.

GC B cell and GC Tfh cell frequencies followed a normal distribution. Therefore, paired Student’s t tests were used for evaluating differences between samples obtained from the same animal at different time points, and unpaired Student’s t tests were used for evaluating differences among groups. Two-tailed Pearson correlation tests were used to assess correlations between GC B cell frequencies or GC Tfh cell frequencies and BG505 nAb titers or BG505 binding titers. All Ab titers were log transformed. Pearson r values and two-tailed p values are reported unless otherwise stated. Not significant is denoted as “ns.” All nAb titer data panels show geometric mean titers with geometric standard deviation. All ELISA Ab data panels show geometric mean titers with geometric standard deviation. All cell frequency data panels show mean frequency and standard deviation. Sample sizes (n) are provided in the respective figure legends.

### Data and Software Availability

We provide the great majority of neutralization and ELISA binding data of all NHPs in the study in Table S1, which is provided as an Excel file to allow for easy independent analysis.

## Author Contributions

The immunogen working group of The Scripps Research Institute (TSRI) and Center for HIV/AIDS Vaccine Immunology and Immunogen Discovery, consisting of W.R.S., A.B.W., I.A.W., R.T.W., S.C., and D.R.B., designed the immunization study and laid out the experimental strategy with the assistance of M.P., C.H.-D., D.S., S.T.B., and D.H.B. C.A.C., G.O., A.T.d.l.P., S.W.T., A.B.W., and R.W.S. designed and produced BG505 SOSIP.664, BG505 SOSIP.v4.1, and BG505 SOSIP.v5.2 trimers for the study. S.K.S., J.G., and R.T.W. designed and produced BG505 NFL trimers for the study. D.W.K., J.M.S., and W.R.S. designed and produced BG505 Olio6, BG505 Olio6 CD4-KO, and BG505 MD39 CD4-KO trimers for the study and produced BG505 SOSIP.664 for one liposome group. T.T. and D.J.I. performed and oversaw the conjugation and quality control of BG505 liposomes. D.G.C., A.V.B., S.C., D.J.I., and G.S. performed and oversaw the Evans Blue lymphatic drainage study. J.P.N. and D.H.B. oversaw all rhesus macaque immunizations, including sample acquisition, processing, storage, and distribution. J.L., A.C., and D.H.B. performed and oversaw flow cytometry of fine needle aspirates (FNAs) and ELISPOT assays. C.H.-D. and S.C. performed and oversaw data analysis of lymph node FNA samples by flow cytometry. K.K., A.L.O., and S.C. performed and oversaw His-tag ELISA binding experiments. D.S. and D.R.B. performed and oversaw ELISA binding experiments. C.C.L. and D.C.M. performed and oversaw neutralization experiments at Duke University. M.P., R.B., L.E.M., and D.R.B. designed HIV pseudovirus mutants and performed and oversaw neutralization experiments at TSRI. M.P., C.H.-D., K.M.C., A.M.E., and K.P. performed statistical analysis of data sets. A.M.E. and K.P. provided IT and database support for the study. K.M.C., D.W.K., T.T., D.S., M.P., C.H.-D., D.J.I., R.W.S., W.R.S., A.B.W., I.A.W., R.T.W., D.H.B., S.C., and D.R.B. analyzed data sets and contributed edits to the manuscript. M.P., C.H.-D., S.C., and D.R.B. wrote the manuscript.

## Supplemental Information

Document S1. Figures S1–S7 and Table S2

Table S1. All Animal Data Peak values and time points of characterized immune parameters.

Document S2. Article plus Supplemental Information

## References

[bib1] Binley J.M., Sanders R.W., Clas B., Schuelke N., Master A., Guo Y., Kajumo F., Anselma D.J., Maddon P.J., Olson W.C., Moore J.P. (2000). A recombinant human immunodeficiency virus type 1 envelope glycoprotein complex stabilized by an intermolecular disulfide bond between the gp120 and gp41 subunits is an antigenic mimic of the trimeric virion-associated structure. J. Virol..

[bib2] Chung N.P.Y., Matthews K., Kim H.J., Ketas T.J., Golabek M., de Los Reyes K., Korzun J., Yasmeen A., Sanders R.W., Klasse P.J. (2014). Stable 293 T and CHO cell lines expressing cleaved, stable HIV-1 envelope glycoprotein trimers for structural and vaccine studies. Retrovirology.

[bib3] Crotty S. (2014). T follicular helper cell differentiation, function, and roles in disease. Immunity.

[bib4] de Taeye S.W., Ozorowski G., Torrents de la Peña A., Guttman M., Julien J.-P., van den Kerkhof T.L.G.M., Burger J.A., Pritchard L.K., Pugach P., Yasmeen A. (2015). Immunogenicity of stabilized HIV-1 envelope trimers with reduced exposure of non-neutralizing epitopes. Cell.

[bib5] de Taeye S.W., Moore J.P., Sanders R.W. (2016). HIV-1 envelope trimer design and immunization strategies to induce broadly neutralizing antibodies. Trends Immunol..

[bib6] deCamp A., Hraber P., Bailer R.T., Seaman M.S., Ochsenbauer C., Kappes J., Gottardo R., Edlefsen P., Self S., Tang H. (2014). Global panel of HIV-1 Env reference strains for standardized assessments of vaccine-elicited neutralizing antibodies. J. Virol..

[bib7] Feng Y., Tran K., Bale S., Kumar S., Guenaga J., Wilson R., de Val N., Arendt H., DeStefano J., Ward A.B., Wyatt R.T. (2016). Thermostability of well-ordered HIV spikes correlates with the elicitation of autologous tier 2 neutralizing antibodies. PLoS Pathog..

[bib8] Gonzalez-Figueroa P., Roco J.A., Vinuesa C.G. (2017). Germinal center lymphocyte ratios and successful HIV vaccines. Trends Mol. Med..

[bib9] Guenaga J., Dubrovskaya V., de Val N., Sharma S.K., Carrette B., Ward A.B., Wyatt R.T. (2015). Structure-guided redesign increases the propensity of HIV Env to generate highly stable soluble trimers. J. Virol..

[bib10] Havenar-Daughton C., Carnathan D.G., Torrents de la Peña A., Pauthner M., Briney B., Reiss S.M., Wood J.S., Kaushik K., van Gils M.J., Rosales S.L. (2016). Direct probing of germinal center responses reveals immunological features and bottlenecks for neutralizing antibody responses to HIV Env trimer. Cell Rep..

[bib11] Havenar-Daughton C., Lee J.H., Crotty S. (2017). Tfh cells and HIV bnAbs, an immunodominance model of the HIV neutralizing antibody generation problem. Immunol. Rev..

[bib12] Haynes B.F., Gilbert P.B., McElrath M.J., Zolla-Pazner S., Tomaras G.D., Alam S.M., Evans D.T., Montefiori D.C., Karnasuta C., Sutthent R. (2012). Immune-correlates analysis of an HIV-1 vaccine efficacy trial. N. Engl. J. Med..

[bib13] Hessell A.J., Malherbe D.C., Pissani F., McBurney S., Krebs S.J., Gomes M., Pandey S., Sutton W.F., Burwitz B.J., Gray M. (2016). Achieving potent autologous neutralizing antibody responses against tier 2 HIV-1 viruses by strategic selection of envelope immunogens. J. Immunol..

[bib14] Hu J.K., Crampton J.C., Cupo A., Ketas T., van Gils M.J., Sliepen K., de Taeye S.W., Sok D., Ozorowski G., Deresa I. (2015). Murine antibody responses to cleaved soluble HIV-1 envelope trimers are highly restricted in specificity. J. Virol..

[bib15] Ingale J., Stano A., Guenaga J., Sharma S.K., Nemazee D., Zwick M.B., Wyatt R.T. (2016). High-density array of well-ordered HIV-1 spikes on synthetic liposomal nanoparticles efficiently activate B cells. Cell Rep..

[bib16] Klein S.L., Flanagan K.L. (2016). Sex differences in immune responses. Nat. Rev. Immunol..

[bib17] Landais E., Huang X., Havenar-Daughton C., Murrell B., Price M.A., Wickramasinghe L., Ramos A., Bian C.B., Simek M., Allen S. (2016). Broadly neutralizing antibody responses in a large longitudinal sub-Saharan HIV primary infection cohort. PLoS Pathog..

[bib18] Li M., Gao F., Mascola J.R., Stamatatos L., Polonis V.R., Koutsoukos M., Voss G., Goepfert P., Gilbert P., Greene K.M. (2005). Human immunodeficiency virus type 1 env clones from acute and early subtype B infections for standardized assessments of vaccine-elicited neutralizing antibodies. J. Virol..

[bib19] Locci M., Havenar-Daughton C., Landais E., Wu J., Kroenke M.A., Arlehamn C.L., Su L.F., Cubas R., Davis M.M., Sette A., International AIDS Vaccine Initiative Protocol C Principal Investigators (2013). Human circulating PD-1+CXCR3-CXCR5+ memory Tfh cells are highly functional and correlate with broadly neutralizing HIV antibody responses. Immunity.

[bib20] Mascola J.R., Montefiori D.C. (2010). The role of antibodies in HIV vaccines. Annu. Rev. Immunol..

[bib21] Mascola J.R., D’Souza P., Gilbert P., Hahn B.H., Haigwood N.L., Morris L., Petropoulos C.J., Polonis V.R., Sarzotti M., Montefiori D.C. (2005). Recommendations for the design and use of standard virus panels to assess neutralizing antibody responses elicited by candidate human immunodeficiency virus type 1 vaccines. J. Virol..

[bib22] McCoy L.E., van Gils M.J., Ozorowski G., Messmer T., Briney B., Voss J.E., Kulp D.W., Macauley M.S., Sok D., Pauthner M. (2016). Holes in the glycan shield of the native HIV envelope are a target of trimer-elicited neutralizing antibodies. Cell Rep..

[bib23] McGuire A.T., Dreyer A.M., Carbonetti S., Lippy A., Glenn J., Scheid J.F., Mouquet H., Stamatatos L. (2014). HIV antibodies. Antigen modification regulates competition of broad and narrow neutralizing HIV antibodies. Science.

[bib24] Plotkin S.A. (2010). Correlates of protection induced by vaccination. Clin. Vaccine Immunol..

[bib25] Pugach P., Ozorowski G., Cupo A., Ringe R., Yasmeen A., de Val N., Derking R., Kim H.J., Korzun J., Golabek M. (2015). A native-like SOSIP.664 trimer based on an HIV-1 subtype B env gene. J. Virol..

[bib26] Richman D.D., Wrin T., Little S.J., Petropoulos C.J. (2003). Rapid evolution of the neutralizing antibody response to HIV type 1 infection. Proc. Natl. Acad. Sci. USA.

[bib27] Sanders R.W., Moore J.P. (2017). Native-like Env trimers as a platform for HIV-1 vaccine design. Immunol. Rev..

[bib28] Sanders R.W., Vesanen M., Schuelke N., Master A., Schiffner L., Kalyanaraman R., Paluch M., Berkhout B., Maddon P.J., Olson W.C. (2002). Stabilization of the soluble, cleaved, trimeric form of the envelope glycoprotein complex of human immunodeficiency virus type 1. J. Virol..

[bib29] Sanders R.W., Derking R., Cupo A., Julien J.-P., Yasmeen A., de Val N., Kim H.J., Blattner C., de la Peña A.T., Korzun J. (2013). A next-generation cleaved, soluble HIV-1 Env trimer, BG505 SOSIP.664 gp140, expresses multiple epitopes for broadly neutralizing but not non-neutralizing antibodies. PLoS Pathog..

[bib30] Sanders R.W., van Gils M.J., Derking R., Sok D., Ketas T.J., Burger J.A., Ozorowski G., Cupo A., Simonich C., Goo L. (2015). HIV-1 VACCINES. HIV-1 neutralizing antibodies induced by native-like envelope trimers. Science.

[bib31] Sharma S.K., de Val N., Bale S., Guenaga J., Tran K., Feng Y., Dubrovskaya V., Ward A.B., Wyatt R.T. (2015). Cleavage-independent HIV-1 Env trimers engineered as soluble native spike mimetics for vaccine design. Cell Rep..

[bib32] Sok D., Pauthner M., Briney B., Lee J.H., Saye-Francisco K.L., Hsueh J., Ramos A., Le K.M., Jones M., Jardine J.G. (2016). A prominent site of antibody vulnerability on HIV envelope incorporates a motif associated with CCR5 binding and its camouflaging glycans. Immunity.

[bib33] Steichen J.M., Kulp D.W., Tokatlian T., Escolano A., Dosenovic P., Stanfield R.L., McCoy L.E., Ozorowski G., Hu X., Kalyuzhniy O. (2016). HIV vaccine design to target germline precursors of glycan-dependent broadly neutralizing antibodies. Immunity.

[bib34] Sun H.-X., Xie Y., Ye Y.-P. (2009). ISCOMs and ISCOMATRIX. Vaccine.

[bib35] Tam H.H., Melo M.B., Kang M., Pelet J.M., Ruda V.M., Foley M.H., Hu J.K., Kumari S., Crampton J., Baldeon A.D. (2016). Sustained antigen availability during germinal center initiation enhances antibody responses to vaccination. Proc. Natl. Acad. Sci. USA.

[bib36] Ueno H., Banchereau J., Vinuesa C.G. (2015). Pathophysiology of T follicular helper cells in humans and mice. Nat. Immunol..

[bib37] Victora G.D., Nussenzweig M.C. (2012). Germinal centers. Annu. Rev. Immunol..

[bib38] Walker L.M., Simek M.D., Priddy F., Gach J.S., Wagner D., Zwick M.B., Phogat S.K., Poignard P., Burton D.R. (2010). A limited number of antibody specificities mediate broad and potent serum neutralization in selected HIV-1 infected individuals. PLoS Pathog..

[bib39] Wei X., Decker J.M., Wang S., Hui H., Kappes J.C., Wu X., Salazar-Gonzalez J.F., Salazar M.G., Kilby J.M., Saag M.S. (2003). Antibody neutralization and escape by HIV-1. Nature.

[bib40] Wu X., Parast A.B., Richardson B.A., Nduati R., John-Stewart G., Mbori-Ngacha D., Rainwater S.M.J., Overbaugh J. (2006). Neutralization escape variants of human immunodeficiency virus type 1 are transmitted from mother to infant. J. Virol..

