# Supplementary material for: Elicitation of Robust Tier 2 Neutralizing Antibody Responses in Nonhuman Primates by HIV Envelope Trimer Immunization Using Optimized Approaches
Source: Immunity. 2017 Jun 20;46(6):1073–1088.e6. doi: 10.1016/j.immuni.2017.05.007 (PMC5483234; doi:10.1016/j.immuni.2017.05.007)
Supplement: Document S1. Figures S1–S7 and Table S2 [file mmc1.pdf]

Immunity, Volume *46*

Supplemental Information

Elicitation of Robust Tier 2 Neutralizing Antibody Responses in Nonhuman Primates by HIV Envelope Trimer Immunization Using Optimized Approaches

Matthias Pauthner, Colin Havenar-Daughton, Devin Sok, Joseph P. Nkolola, Raiza Bastidas, Archana V. Boopathy, Diane G. Carnathan, Abishek Chandrashekar, Kimberly M. Cirelli, Christopher A. Cottrell, Alexey M. Eroshkin, Javier Guenaga, Kirti Kaushik, Daniel W. Kulp, Jinyan Liu, Laura E. McCoy, Aaron L. Oom, Gabriel Ozorowski, Kai W. Post, Shailendra K. Sharma, Jon M. Steichen, Steven W. de Taeye, Talar Tokatlian, Alba Torrents de la Peña, Salvatore T. Butera, Celia C. LaBranche, David C. Montefiori, Guido Silvestri, Ian A. Wilson, Darrell J. Irvine, Rogier W. Sanders, William R. Schief, Andrew B. Ward, Richard T. Wyatt, Dan H. Barouch, Shane Crotty, and Dennis R. Burton

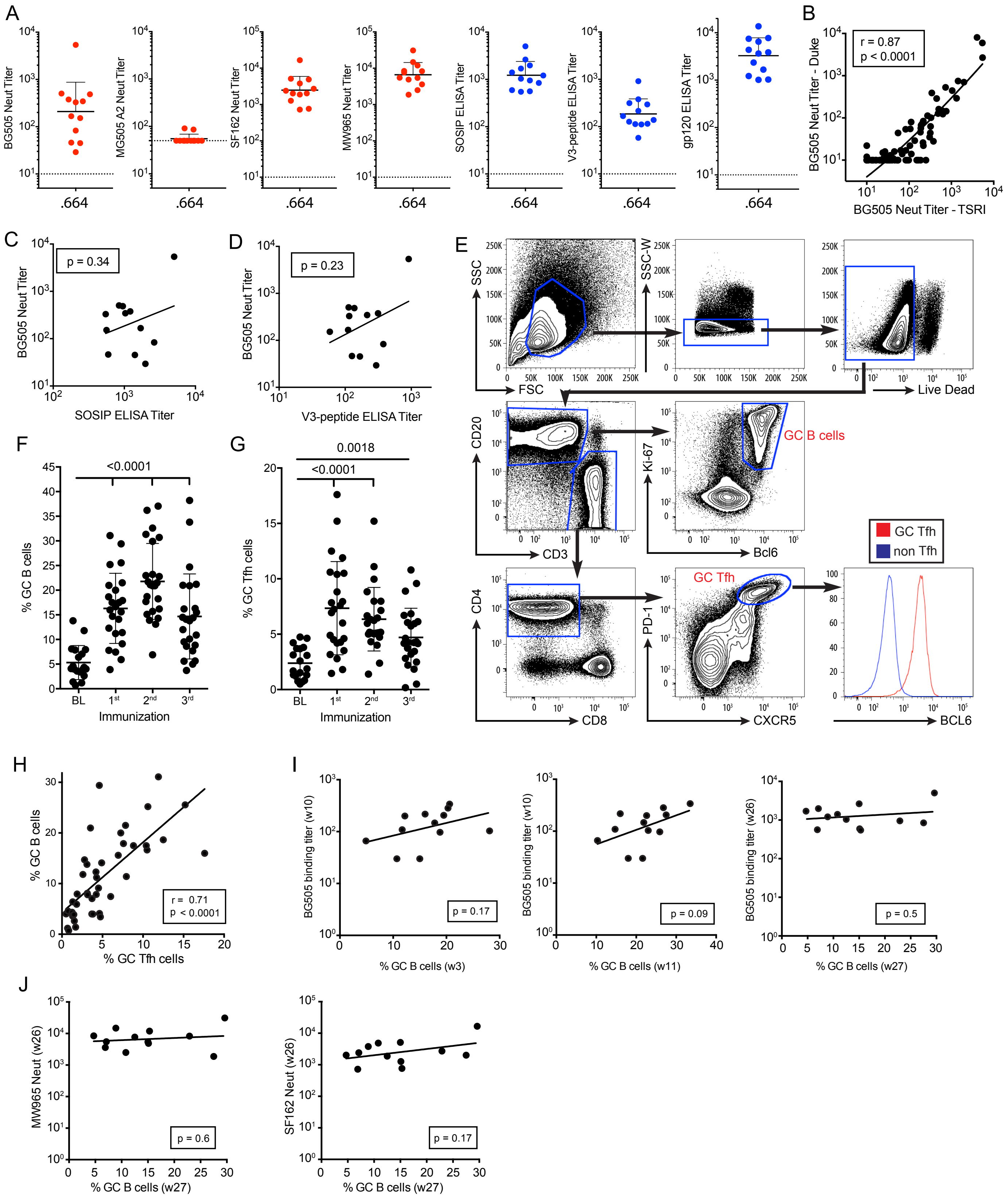

Figure S1: BG505 SOSIP.664 immunized animals. Related to Figure 1.
Antibody and germinal center cell kinetics and correlations.
A) Post 3rd immunization antibody responses of BG505 SOSIP.664 immunized RMs (".664", n = 12). Ab titers of autologous Tier 2 BG505 nAb, Tier 2 MG505 nAb, Tier 1 SF162 nAb, Tier 1 MW965 nAb, BG505 SOSIP binding titer, V3-loop peptide titer, and BG505 gp120 binding titer. Red indicates a neutralization assay, blue indicates an ELISA assay. LOD, limit of detection
B) Correlation of BG505 nAb titers measured at TSRI or Duke University sites. N = 78.

C) Lack of correlation between BG505 SOSIP binding titer and BG505 nAb titer. N = 12.
D) Lack of correlation between V3-loop peptide binding titer and BG505 nAb titer. N = 12.
E) Example flow cytometry gating scheme for identification of GC Tfh cells and GC B cells in LN FNA samples.
F) GC B cell frequencies among total $CD20^+$ B cells from LN FNA samples taken at baseline (BL) and 3 weeks after each immunization. Points represent individual LNs. Mean and SD are shown. N = 24 for each time point.
G) GC Tfh cell frequencies among total $CD4^+$ T cells from LN FNA samples taken at baseline (BL) and 3 weeks after each immunization. Mean and SD are shown. Points represent individual LNs. Mean and SD are shown. N = 24 for each time point.
H) Correlation of GC B cells and GC Tfh cells in LN FNA samples before and after the 1st immunization. Points represent individual LNs. N = 48.
I) Lack of correlation between BG505 IgG titers and GC B cell frequency (calculated as the mean of the two inguinal draining LNs per animal) after the 1st (left), 2nd (center), or 3rd (right) immunization. N = 12 for all panels.
J) Lack of correlation between Tier 1 nAb and GC B cell frequency (calculated as the mean of the two inguinal draining LNs per animal) after the 3rd immunization. Tier 1 MW965 (left) and Tier 1 SF162 (right). N = 12 for both panels.
All nAb titer and ELISA binding Ab data panels show geometric mean titers with geometric SD. All cell frequency data panels show mean and SD.

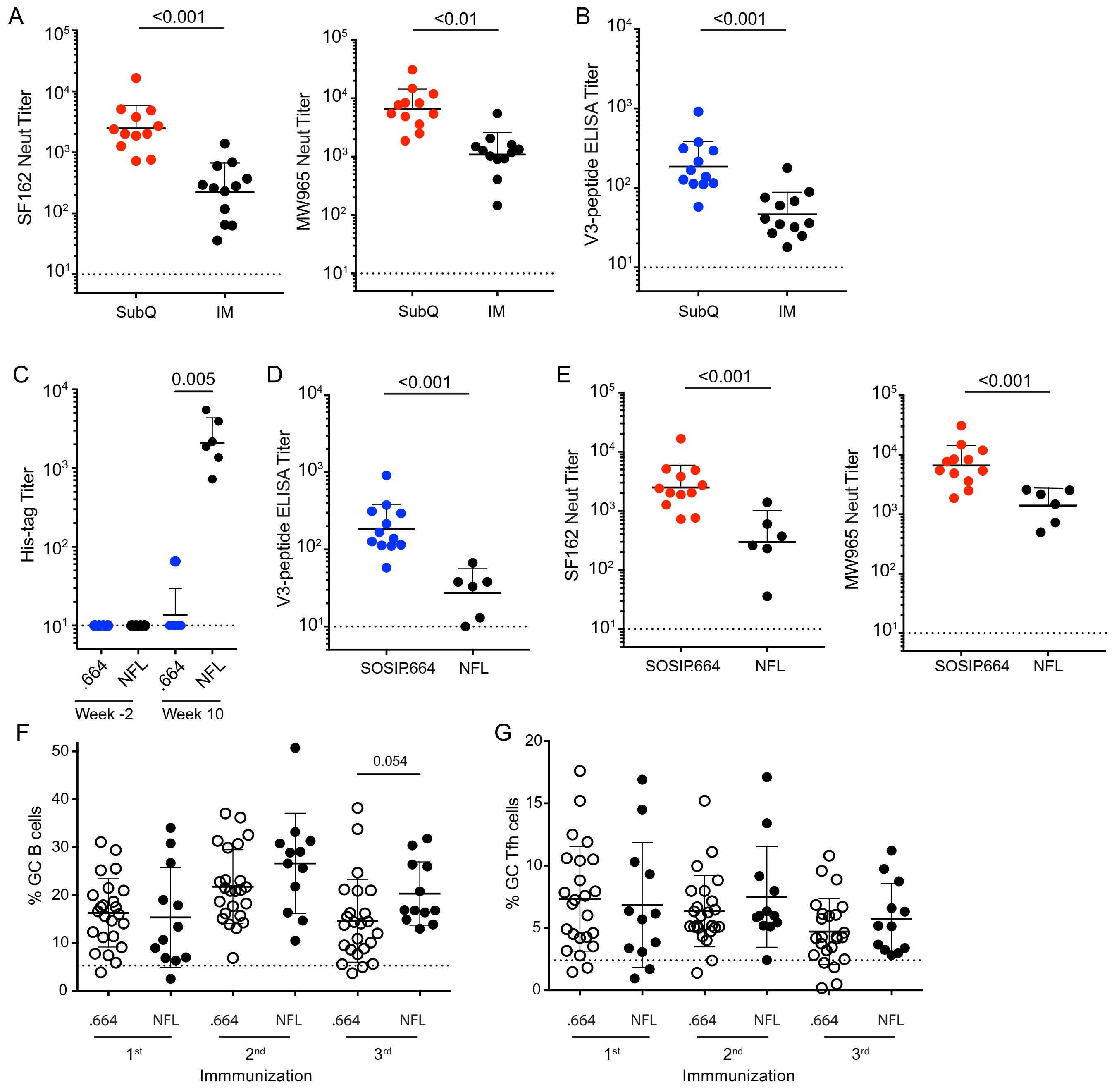

Figure S2: Effects of subcutaneous versus intramuscular immunization route and comparison of NFL and SOSIP Env trimers. Related to Figure 2.

(A-B) Comparison of subcutaneous (s.c. or SubQ) and intramuscular (i.m. or IM) immunization routes

A) Tier 1 nAb responses of s.c. and i.m. BG505 SOSIP.664 immunized RMs, 2 weeks after the 3rd immunization. Week 26, N = 12.

B) V3-loop peptide IgG titer of s.c. and i.m. BG505 SOSIP.664 immunized RMs, 2 weeks after the 3rd immunization. Week 26, N = 12.

(C-F) Comparison of BG505 SOSIP.664 and BG505 NFL s.c. immunizations.

C) His-tag peptide IgG titer of BG505 SOSIP.664 (N = 6) and BG505 NFL (N = 6) immunized RMs before the 1st immunization (Week -2) and 2 weeks after the 2nd immunization (Week 10).

D) V3-loop peptide IgG titer of BG505 SOSIP.664 (N = 12) and BG505 NFL (N = 6) immunized RMs, 2 weeks after the 3rd immunization. Week 26.

E) Tier 1 nAb responses of BG505 SOSIP.664 (N = 12) and BG505 NFL (N = 6) immunized RMs, 2 weeks after the 3rd immunization. Week 26.

F) GC B cell frequencies among CD20^{+} B cells from LN FNA samples taken 3 weeks after each immunization. N = 12 or 24 per time point.

G) GC Tfh cell frequencies among $CD4^+$ T cells from LN FNA samples taken 3 weeks after each immunization. N = 12 or 24 per time point.

All nAb titer and ELISA binding Ab data panels show geometric mean titers with geometric SD. All cell frequency data panels show mean and SD.

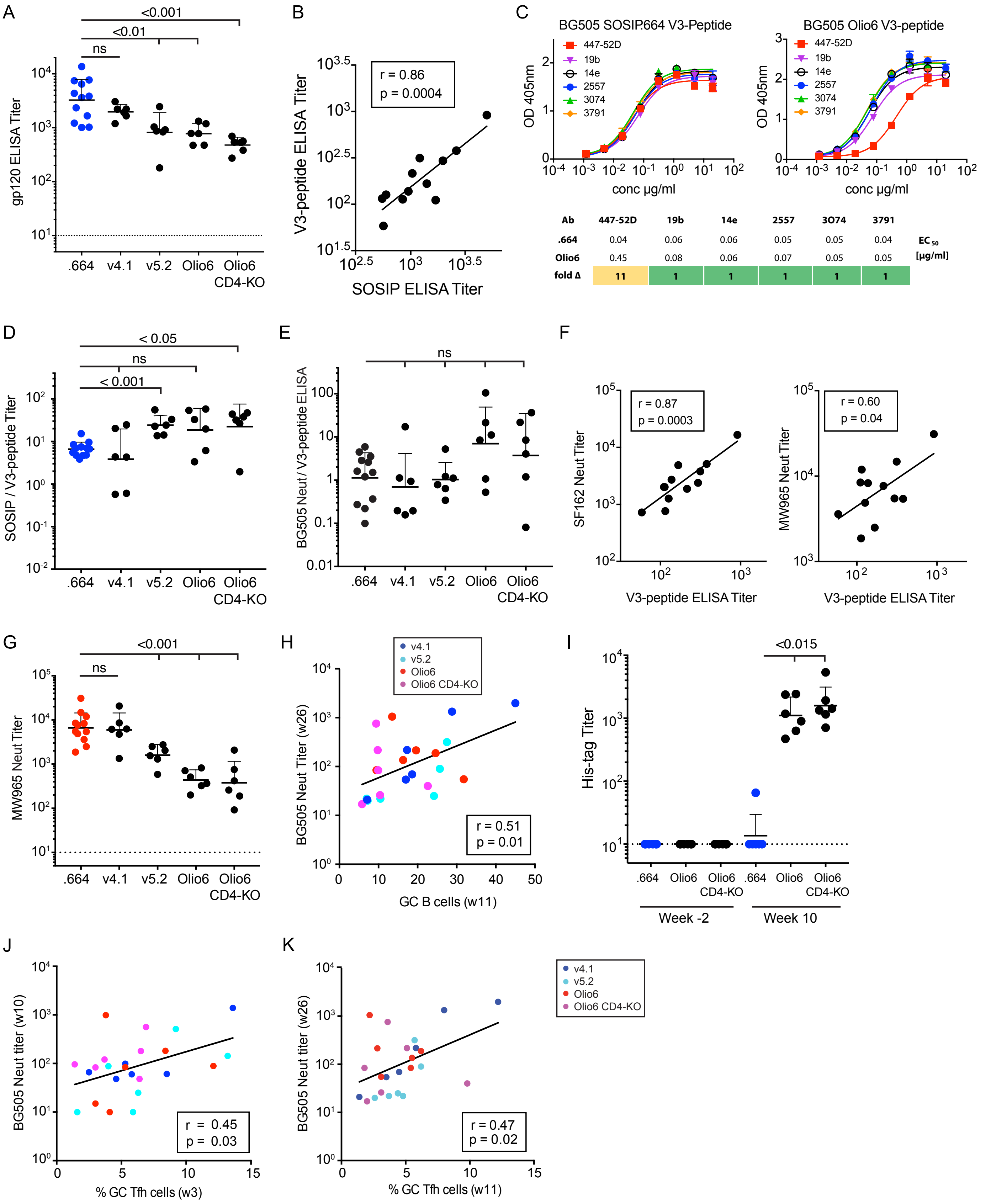

Figure S3: Immunization with stabilized Env trimers. Related to Figure 3.
Modified BG505 SOSIP and BG505 Olio6 constructs were compared to the BG505 SOSIP.664 immunogen. Throughout the figure immunogen names are abbreviated as follows: BG505 SOSIP.664 (.664), BG505 SOSIP.v4.1 (v4.1), BG505 SOSIP.v5.2 (v5.2), BG505 SOSIP Olio6 (Olio6), and BG505 SOSIP Olio6 CD4-KO (Olio6 CD4-KO). ns, non-significant.

A) BG505 gp120 binding IgG titer 2 weeks after the 3rd immunization. Week 26, N = 6 or 12.
B) Correlation of V3-loop binding IgG titers and BG505 SOSIP binding IgG titers in BG505 SOSIP.664 immunized macaques two weeks after the 3rd immunization. Week 26, N = 12.
C) BG505 WT and Olio6 V3-peptide antigenicity comparison. A panel of V3-loop directed Abs were tested for their ability to bind BG505 WT (.664) and Olio 6 V3-peptides. EC_{50} binding titers and fold-difference in titers between WT and Olio6 peptides are shown below.
D) Ratio of BG505 SOSIP to BG505 V3-peptide titers in immunized RMs two weeks after the 3rd immunization. Week 26, N = 6 or 12
E) The ratio of BG505 nAb titer to V3-peptide binding IgG titer after the 3rd immunization. Week 26, N = 6 or 12
F) Correlation of V3-loop binding IgG titers and (Left) Tier 1 SF162 nAb titers and (Right) Tier 1 MW965 nAb titers after the 3rd immunization. BG505 SOSIP.664 immunized RMs. Week 26, N = 12.
G) Tier 1 MW965 nAb titers after the 3rd immunization. Week 26, N = 6 or 12.
H) Correlation of GC B cell frequencies (week 11) and BG505 nAb titers (week 26) in RMs immunized with stabilized Env trimers. Immunogen groups are color coded. N = 24.
I) His-tag peptide IgG titer of BG505 SOSIP.664 (N = 6), BG505 Olio6 (N = 6), and BG505 Olio6 CD4-KO (N = 6) immunized RMs before the 1st immunization (Week -2) and 2 weeks after the 2nd immunization (Week 10).
J) Correlation of GC Tfh cell frequencies (week 3) and BG505 nAb titers (week 10) in RMs immunized with stabilized Env trimers. Immunogen groups are color coded as in H. N = 24.
K) Correlation of GC Tfh cell frequencies (week 11) and BG505 nAb titers (week 26) in RMs immunized with stabilized Env trimers. Immunogen groups are color coded as in H. N = 24.
All nAb titer and ELISA binding Ab data panels show geometric mean titers with geometric SD.

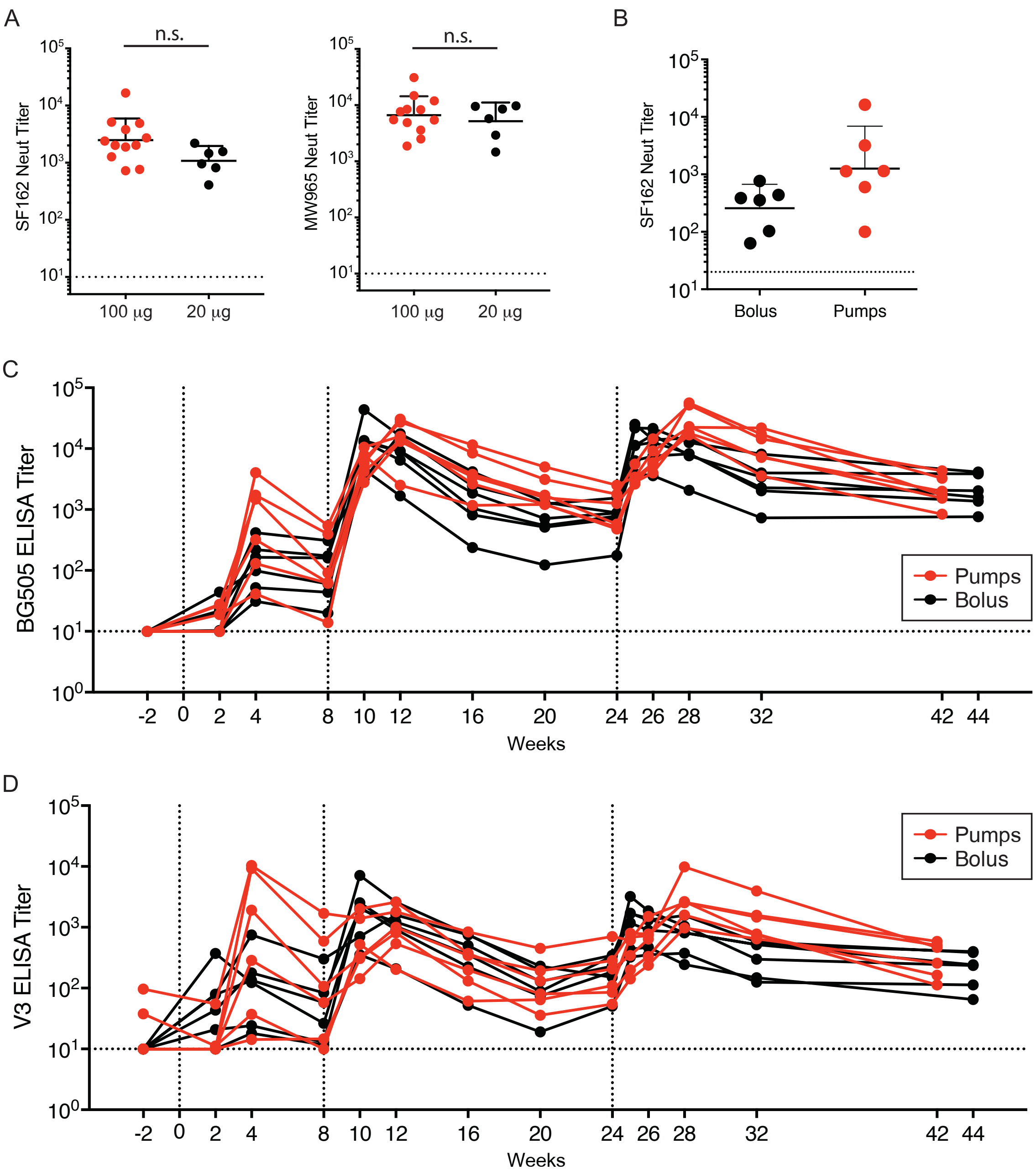

Figure S4: Effects of vaccine dose and continuous immunogen delivery. Related to Figure 4.

A) Tier 1 SF162 nAb titers (Left) and Tier 1 MW965 nAb titers (Right) two weeks after the 3rd immunization in RMs immunized with either 100 µg or 20 µg of BG505 SOSIP.664. Week 26, N = 6 or 12.

(B-E) Comparison of conventional bolus immunization and continuous immunogen delivery

B) Tier 1 SF162 nAb titers two weeks after the end of the third immunization in RMs immunized with BG505 SOSIP.v5.2 either as a conventional bolus injection ('bolus', week 26) or after continuous antigen delivery ('pumps', week 28). N = 6.

C) Kinetics of BG505 binding IgG titers in RMs immunized with BG505 SOSIP.v5.2 either as a conventional bolus injection ('bolus') or after continuous antigen delivery ('pumps'). Samples were analyzed in two batches, week -2 through week 20 and week 24 through week 44. N = 6 per group.

D) Kinetics of V3-loop peptide binding IgG titers in RMs immunized with BG505 SOSIP.v5.2 either as a conventional bolus injection ('bolus') or after continuous antigen delivery ('pumps'). Samples were analyzed in two batches, week -2 through week 20 and week 24 through week 44. N = 6 per group.

All nAb titer data panels show geometric mean titers with geometric SD.

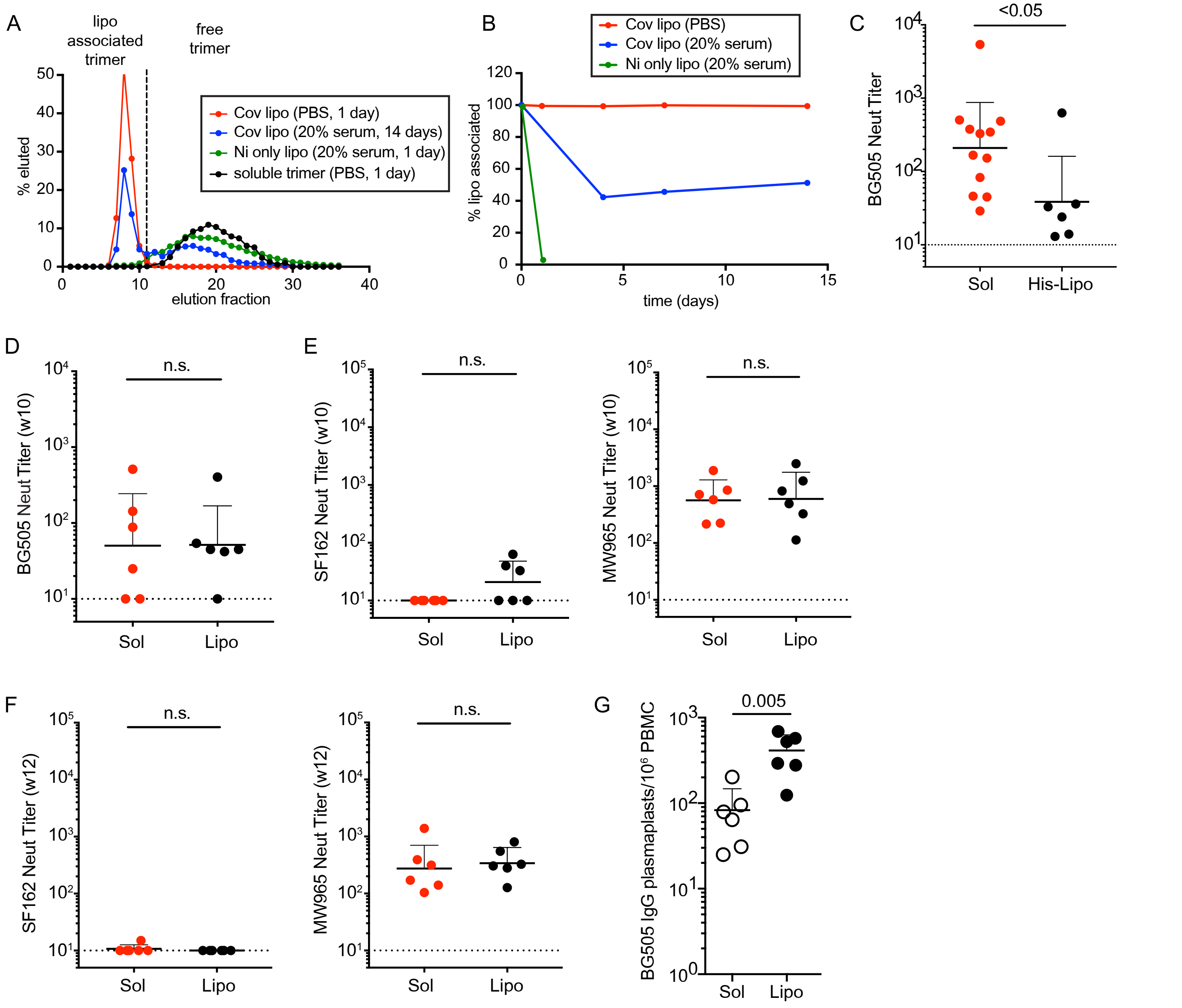

Figure S5: Effects of Env trimer presentation on liposomes. Related to Figure 5.

(A-B) Liposome conjugation and stability testing.

A) Stability of covalent liposomes in rhesus serum. Representative SEC elution curves. Liposome associated trimers are in elution fractions to the left of the dashed vertical line. Free soluble trimers are in elution fractions to the right of the dashed vertical line. Non-covalent liposomes without MPB (Ni only) release the majority of bound trimers after only 1 day in 20% rhesus serum.

B) Covalent liposomes maintain ~40-50% of trimers after 14 days in serum. Data is representative of multiple experiments.

C) RMs immunized with soluble BG505 SOSIP.664 or His-tag-Ni linked BG505 SOSIP.664 liposomes (‘His-Lipo.’). BG505 nAb titers after the 3rd immunization. Week 26, N = 12 or 6.

(D-G) RMs immunized with soluble BG505 Olio6 CD4-KO (‘sol’) or covalently conjugated BG505 Olio6 CD4-KO liposomes (‘lipo’).

D) BG505 nAb titers two weeks after the 2nd immunization. Week 10, N = 6.

E) Tier 1 SF162 nAb titers (Left) and Tier 1 MW965 nAb titers (Right) two weeks after the 2nd immunization. Week 10, N = 6.

F) Tier 1 SF162 nAb titers (Left) and Tier 1 MW965 nAb titers (Right) four weeks after the 2nd immunization. Week 12, N = 6.

G) BG505 SOSIP.664-specific blood plasmablasts 1 week after the 2nd immunization. Week 9, N = 6

All nAb titer data panels show geometric mean titers with geometric SD. All cell frequency data panels show mean and SD.

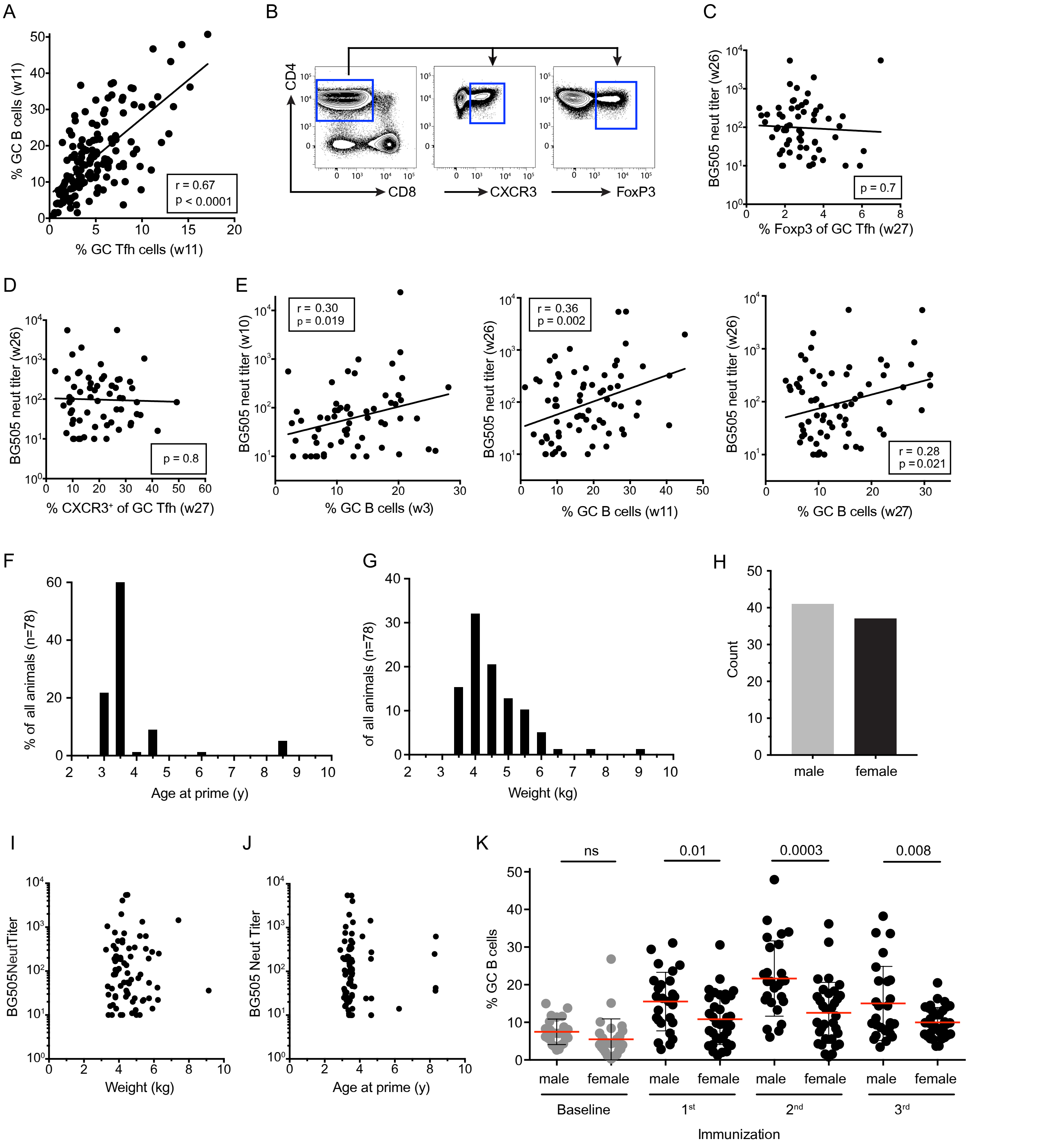

Figure S6: All animal analysis. Related to Figure 6.

A) Correlation analysis of GC B cells and GC Tfh cells in LN FNA samples before and after the 1st immunization. Points represent individual LNs. N = 144.

B) Reference flow cytometry gating used to guide gating of CXCR3+ GC Tfh cells and FoxP3+ GC Tfh cells shown in Figure 6D. RM LN cells showing two separate subsets of CD4+ T cells, CXCR3+ CD4 T cells (middle panel) and FoxP3+ CD4 Treg cells (right panel).

C) Lack of correlation between BG505 nAb titer and the frequency of FoxP3+ GC Tfh cells, shown in Figure 6D-E, after the 3rd immunization. N = 72.

D) Lack of correlation between BG505 nAb titer and the frequency of CXCR3+ GC Tfh cells, shown in Figure

6D-E, after the 3rd immunization. N = 72.
E) GC B cell frequencies (mean of 2 draining LNs per animal) predicts BG505 nAb titers at multiple time points. Line shows linear regression. Pump animals are excluded, due to different GC B cell and neut kinetics. N = 72.
F) Age (in years) of all animals in the study. N = 78.
G) Weight (in kilograms) of all animals in the study. N = 78.
H) Gender of all animals in the study. N = 78.
I) Lack of correlation between weight and BG505 nAb titer. N= 78.
J) Lack of correlation between age and BG505 nAb titer. N = 78.
K) GC B cell frequencies of total $CD20^+$ B cells in male and female RMs immunized with BG505 SOSIP.664 (SOSIP.664 s.c., SOSIP.664 i.m., and SOSIP.664 20 μg groups included). N = 120 per time point.
All nAb titer data panels show geometric mean titers with geometric SD. All cell frequency data panels show mean and SD.

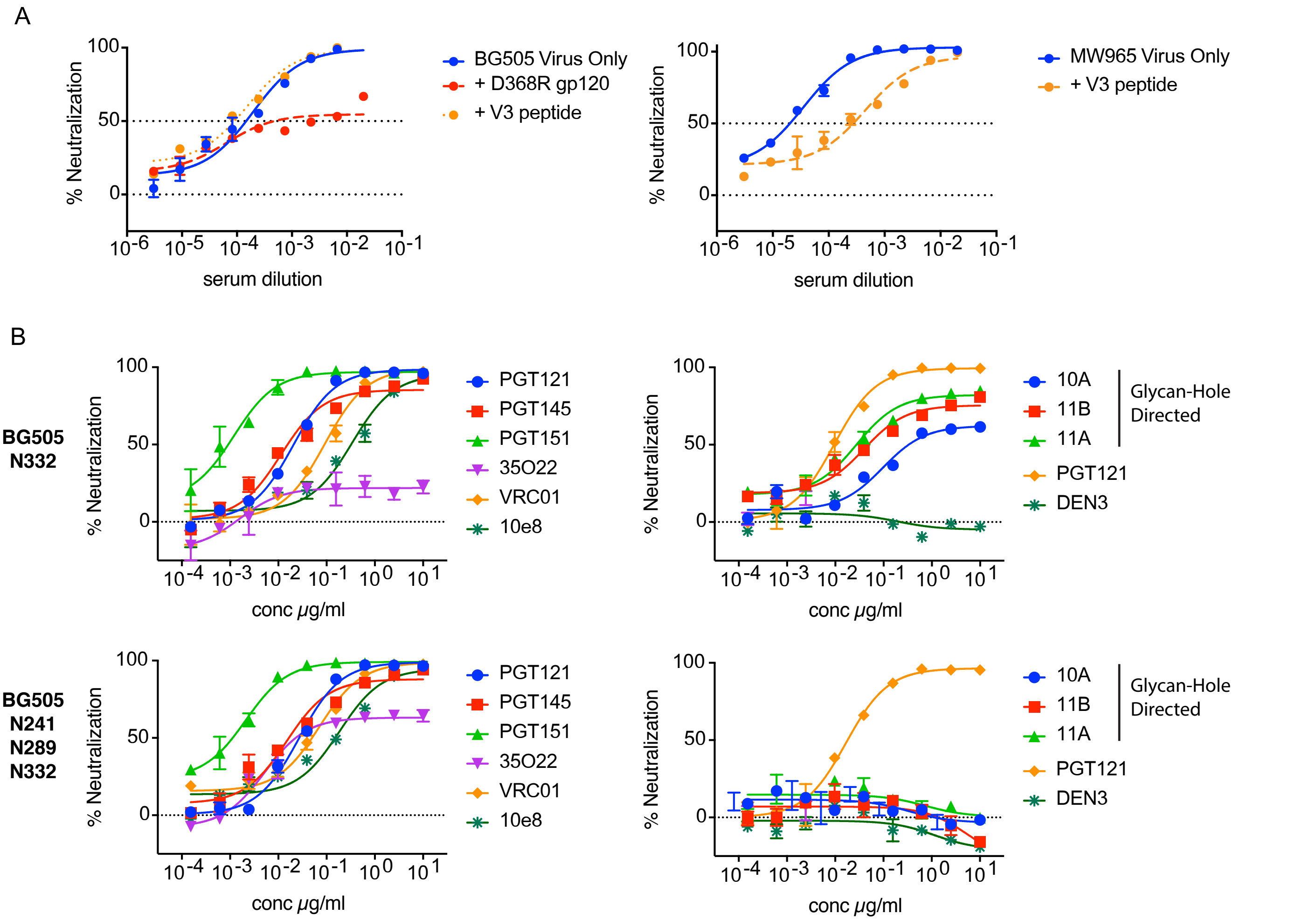

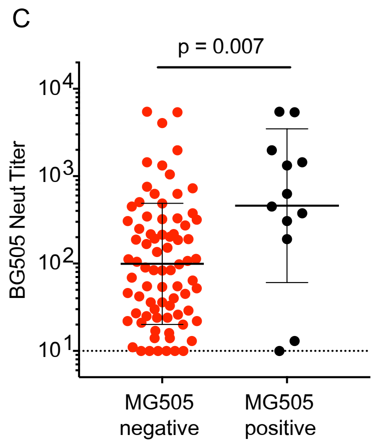

D

V1-loop start

**BG505**	PLCVTLQCTN_VTN_NITDDMRG
**MG505 A2**	PLCVTLQCTN_VTN_NITDDMRG
**MG505 H3**	PLCVTLQCTNNVTNANITDDMRG

E / F

	MG505 H3 CTNNVTNA	MG505 H3 CTN_VTN_	MG505 A2 CTN_VTN_	MG505 A2 CTNNVTN_	MG505 A2 CTNAVTN_	MG505 A2 CTNNVTNA	
**12-084**	73	2	333	2	2	2	.664
**12-137**	<30		49	> 2	2	1	v4.1
**12-046**	<30	> 85	1345	9	7	10	v4.1
**12-143**	<30		<30				Olio6
**12-147**	<30		<30				Olio6 CD4-KO
**12M169**	<30		120	1	1	2	v5.2/Pump
**11M088**	<30		800	4	2	7	v5.2/Pump
**0N7**	<30		318	3	2	2	.664 Liposomes
**4O9**	<30	> 435	2919	97	73	97	Olio6 CD4-KO Lipo

Fold increase compared to H3 WT (E)

Fold decrease compared to A2 WT (F)

Figure S7: nAb mapping and breadth. Related to Figure 7.
A) Example BG505 (Left) or Tier 1 MW965 (Right) neutralization curves from epitope mapping experiments. Neutralization assays were performed in the presence of either linear BG505 V3-peptide (yellow) or BG505 D368R gp120 (red).
B) BG505 mutant-virus antigenic verification, shown exemplary for BG505 S241N P291S N332. BG505 N332 (upper panel) and BG505 S241N P291S N332 (lower panel) were tested in neutralization experiments against

bnAbs and non-neutralizing antibodies directed to various epitopes, to confirm intact antigenicity. Rabbit nAbs 10A, 11A and 11B that target the BG505 N241/N289 glycan hole (McCoy et al., 2016), were tested to confirm steric obstruction of glycan hole epitopes. DEN3 was included as a negative control.
C) BG505 nAb titers in immunized RMs that either did (MG505 positive, black) or did not (MG505 negative, red) neutralize MG505 A2 pseudovirus.
D-F) MG505 neutralization epitope mapping.
D) The beginning of the respective V1-loop sequences are shown. Strand A is highlighted in green for reference. Mutant MG505 H3 (E) and A2 (F) pseudoviruses that had amino acids knocked-in or deleted (shown in red) were tested for neutralization by week 26 sera of high BG505-neutralizer RMs. Shown is the fold difference in nAb titers between mutant and WT viruses, as indicated. The mutant viruses are based on two insertions in the MG505 H3 V1-loop that are not present in MG505 A2 and BG505.
All nAb titer data panels show geometric mean titers with geometric SD.

Table S1: All animal data table. Related to Figure 6.
Peak values and time point of characterized immune parameters for all animals examined in the study.

Provided as an Excel file for download.

Table S2: All groups immunogen overview. Related to Figure 6.
Overview of all experimental groups. Listed are all immunogens, their respective production cell lines, purification method, protein-tags (if present) and detailed immunization parameters.

Group	Immunogen	Cell line	Purification	Tag	Ag Presentation	Route	Total Dose	# of animals
BG505 SOSIP.664	BG505 SOSIP.664	293	2G12	-	Soluble	SubQ	100 ug	12
BG505 SOSIP.664 20ug	BG505 SOSIP.664	293	2G12	-	Soluble	SubQ	20 ug	6
BG505 SOSIP.664 IM	BG505 SOSIP.664	293	2G12	-	Soluble	IM	100 ug	6
BG505 SOSIP.664 IM GMP	BG505 SOSIP.664 CHO	CHO	2G12	-	Soluble	IM	100 ug	6
BG505 SOSIPv4.1	BG505 SOSIPv4.1	293	2G12	-	Soluble	SubQ	100 ug	6
BG505 SOSIPv5.2	BG505 SOSIPv5.2	293	PGT145	-	Soluble	SubQ	100 ug	6
BG505 NFL	BG505 NFL	293	Lectin/NS	His	Soluble	SubQ	100 ug	6
BG505 Olio6	BG505 Olio6	293	His	His	Soluble	SubQ	100 ug	6
BG505 Olio6-CD4KO	BG505 Olio6 CD4-KO	293	His	His	Soluble	SubQ	100 ug	6
Pumps	BG505 SOSIPv5.2	293	PGT145	-	Soluble	SubQ	200 ug	6
Lipo SOSIP.664, His-Ni	BG505 SOSIP.664	293	His	His	Liposome	SubQ	100 ug	6
Lipo BG505 Olio6-CD4KO covalent	BG505 Olio6 CD4-KO	293	His	His	Liposome	SubQ	100 ug	6

NS = negative selection by F105 Ab
His = Histidine-tag
